# Total Syntheses of Sappanin‐Based Natural Products Enabled by Pattern Recognition Analysis and Brominative Aromatization

**DOI:** 10.1002/asia.202500886

**Published:** 2025-10-16

**Authors:** Wei‐Ting Zhao, Jing‐Yu Liang, Yen‐Ku Wu

**Affiliations:** ^1^ Department of Applied Chemistry and Center for Emergent Functional Matter Science National Yang Ming Chiao Tung University 1001 University Road Hsinchu 30010 Taiwan; ^2^ Department of Chemistry Chung Yuan Christian University 200 Zhongbei Road Taoyuan City 320314 Taiwan

**Keywords:** Arylation, Brominative aromatization, Pattern‐recognition analysis, Sappanin, Total synthesis

## Abstract

Described is a concise route to caesappins A and B, urolithin C, and protosappanin A, which share a common sappanin core. An arylation of a cyclic vinylogous ester and a brominative aromatization were applied to construct a privileged intermediate, which was subsequently converted to the target natural products through strategic coupling reactions.

## Graphical abstract

A unified synthetic strategy enables the total syntheses of sappanin‐based natural products from a common privileged intermediate derived from cyclic vinylogous esters.

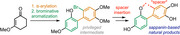

Phenol‐containing natural products bearing biaryl frameworks represent a structurally diverse and biologically significant class of compounds.^[^ ^1^, ^2^ ^]^ Guided by pattern‐recognition analysis,^[^ ^3^ ^]^ we identified that caesappin A (**1**),^[^ ^4^ ^]^ caesappin B (**2**),^[^ ^4^ ^]^ urolithin C (**3**),^[^ ^5^ ^]^ and protosappanin A (**4**)^[^ ^6^ ^]^ share a structural pattern of sappanin yet different “spacers” across the biaryl structure (Scheme 1). Despite their distinct appendages, these compounds present an opportunity for unified synthetic design. We deemed **5** as a synthetic equivalent to the structural pattern and hence a privileged intermediate in the current study. The overall strategy would build on exploiting the privileged intermediate **5** and inserting building blocks into it by leveraging the reactivities of the phenol and the aryl‐Br bond. We envisioned that **5** could be prepared through a brominative aromatization of compound **6**, which is a product of a deprotonative α‐arylation of a cyclic vinylogous ester (CVE).^[^ ^7^, ^8^, ^9^ ^]^ This fragment‐coupling approach features divergent access to sappanin‐based natural products from a common precursor.

**Scheme 1:**
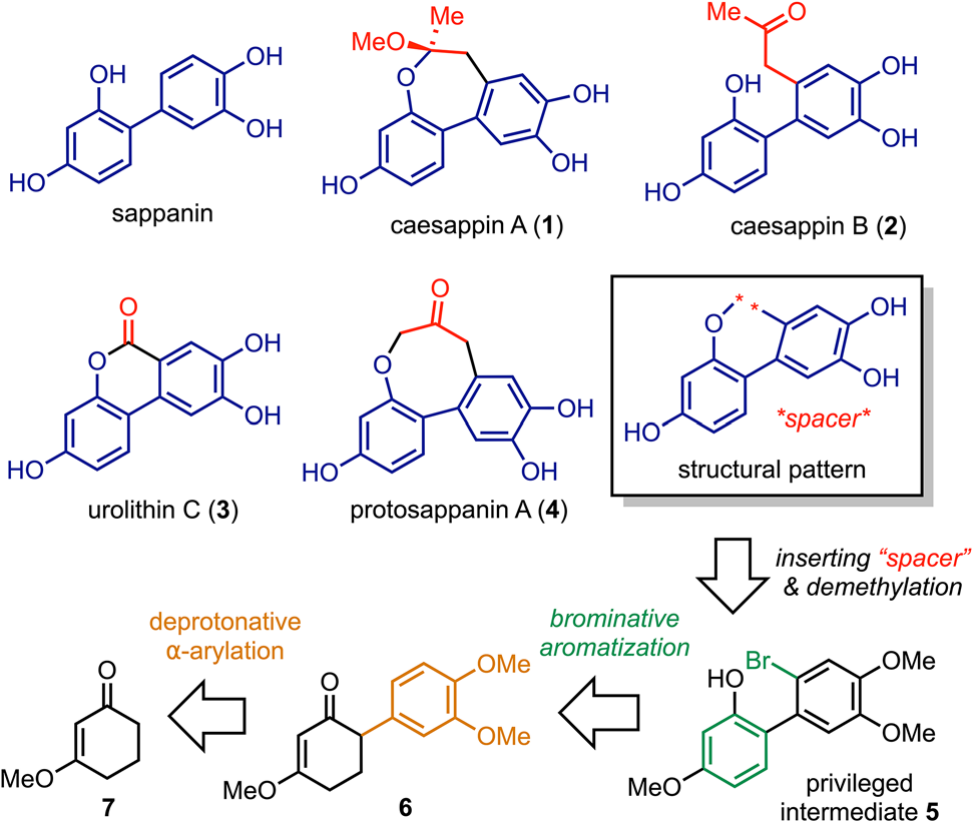
Pattern‐recognition and retrosynthetic analysis.

The task of converting **6** to **5** can be broken down into installing a bromo group to the dimethoxyaryl moiety and realizing a conversion of the CVE core to the substituted phenol. Several research groups have demonstrated the aromatization of six‐membered CVEs under various conditions.^[^ ^10^, ^11^, ^12^, ^13^, ^14^ ^]^ Clive and co‐workers showed that treating 2‐bromo‐substituted CVEs with 1,8‐diazabicyclo[5.4.0]undec‐7‐ene (DBU) furnished the aromatized products.^[^ ^11^ ^]^ This chemistry was particularly suitable for our designed plan and drew us into combining the two bromination events in a single operation (vide infra), followed by the isomerization/elimination with DBU.

Our study commenced with developing a route to the privileged intermediate **5**. The coupling of a CVE, namely 3‐methoxy‐2‐cyclohexenone (**7**), and 4‐bromoveratrole (**8**) proceeded efficiently to give α‐arylated product **6** (Scheme 2). We screened a range of reagents and conditions for the dibromination of **6**. In short, *N*‐bromosuccinimide (NBS, 2.0 equiv.) and I_2_ (10 mol%) emerged as an optimal combination, and the resulting dibrominated product **9** was directly subjected to the Clive protocol^[^ ^11^ ^]^ without column purification, delivering **5** in 60% yield. In the absence of I_2_ catalysis, the bromination of **6** only gave a complex mixture. Mechanistically, Yao and co‐workers have validated an intermediacy of iodine monobromide for NBS/I_2_‐mediated electrophilic aromatic bromination.^[^ ^15^ ^]^ It is noteworthy that the dibromination reaction is of high regioselectivity and efficiency with a stoichiometric amount of NBS. In this regard, Chen and Zhang reported that an aliphatic ketone can direct an iodination reaction of a neighboring aryl group,^[^ ^16^ ^]^ and we wondered if an analogous reactivity mode was operating in our system. When a mixture of 3‐methoxy‐2‐cyclohexenone and veratrole was treated with the optimized conditions (2 equiv. of NBS and 10 mol% of I_2_), a complete conversion of CVE to 2‐bromo‐3‐methoxycyclohex‐2‐en‐1‐one was observed, but only 69% of veratrole was brominated to give 4‐bromoveratrole as judged by proton NMR analysis of the crude mixture. The competitive experiment showed that, to some extent, the bromination at the *α*‐aryl group was facilitated by the proximal carbonyl group embedded in the CVE.^[^ ^16^ ^]^ With the privileged intermediate **5** in hand, the next stage is to insert appropriate building blocks for the total synthesis of sappanin‐based natural products.

**Scheme 2:**
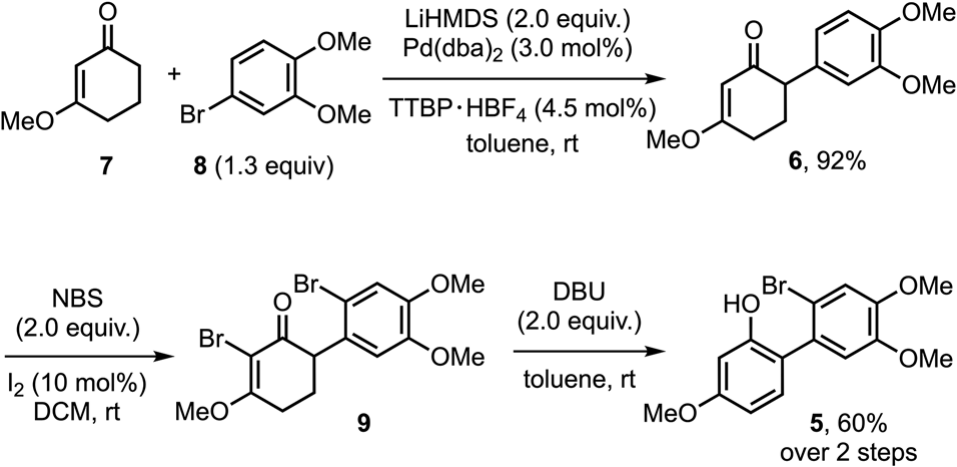
Assembly of the privileged intermediate **5**.

Caesappin A (**1**) features a sappanin‐fused seven‐membered cyclic ketal and displays moderate cytotoxicity against the A549 cell line.^[^ ^4^ ^]^ Following our synthetic analysis, a C3 unit needs to be attached to compound **5** to access **1** (Scheme 1). A conceivable plan is to activate the C(aryl)─Br bond for an acetonylation‐type reaction, followed by an intramolecular ketal formation with the phenol moiety. We surveyed some catalytic cross‐coupling reactions of **5** with allyl nucleophiles, but the starting material remained unconsumed in those trials, possibly due to the interference of the phenol. This issue was addressed by applying a protecting group, and the *O*‐methylation of phenol resulted in **10a** (Scheme 3). Indeed, the palladium‐catalyzed allylation of **10a** with allylstannane proceeded smoothly to give compound **11a** in high yield. Subsequently, the alkenyl moiety was converted to the ketone through Wacker‐Tsuji oxidation,^[^ ^17^, ^18^ ^]^ thus realizing a formal acetonylation of the privileged intermediate. However, attempts to remove methyl groups of **12a** using BBr_3_ or NaSEt were unsuccessful for the synthesis of caesappin B (**2**). We speculated that those highly reactive reagents for the methyl deprotection are detrimental to compound **2**. Accordingly, we changed the methyl protecting groups of phenol to benzyl groups, which can be taken off under relatively neutral conditions. After preparing perbenzylated bromo‐sappanin **10b**, the catalytic allylation of **10b** and the Wacker–Tsuji oxidation of **11b** were uneventfully achieved. To our delight, the hydrogenolysis of **12b** over palladium on charcoal unmasked the phenols, thus furnishing caesappin B (**2**). Applying an acid‐mediated intramolecular ketalization of **2** in methanol led to racemic caesappin A (**1**). Significantly, this work represents the first total syntheses of caesappins A and B.

**Scheme 3:**
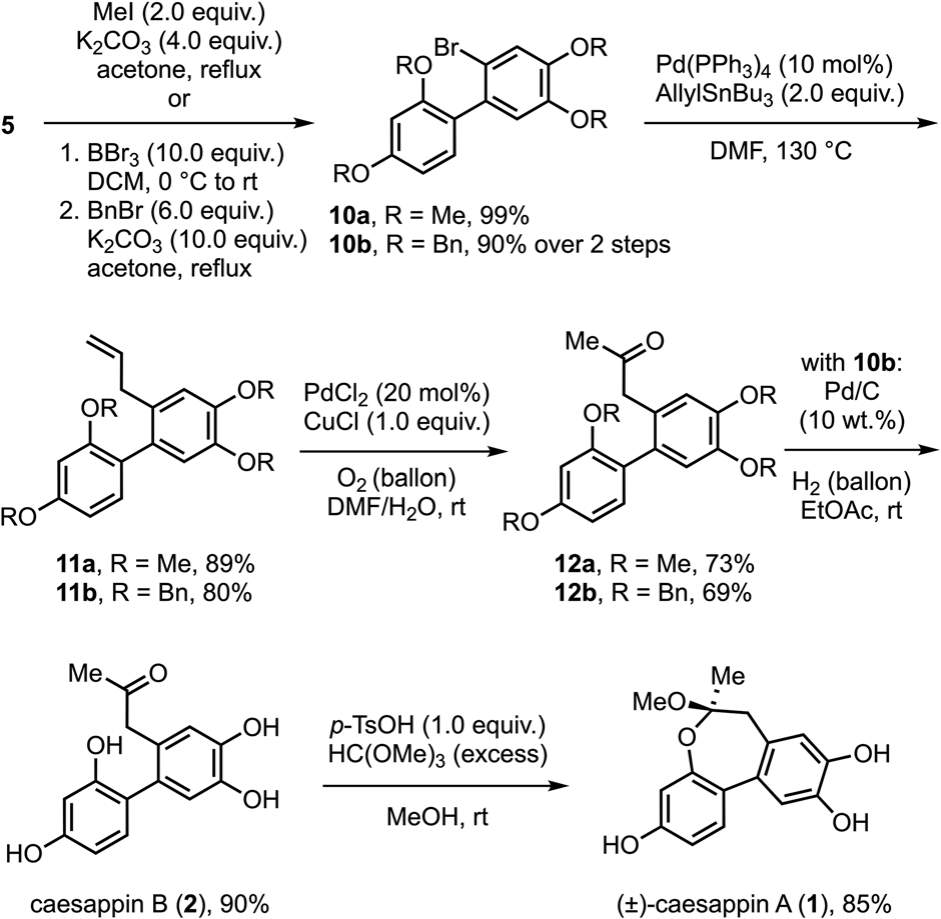
Synthetic route to Caesappins **1** and **2**.

Urolithins possess a structural core of dibenzo‐*α*‐pyrones. Due to their diverse biological activities, many research groups have developed approaches to urolithins.^[^ ^19^, ^20^, ^21^ ^]^ To synthesize urolithin C (**3**), we sought to introduce a C1 unit into the masked privileged intermediate **10a** (Scheme 4). After extensive experimentation, the Rosenmund‐von Braun reaction was used to obtain the cyanation product **13**.^[^ ^22^ ^]^ Subsequently, compound **13** was hydrolyzed under basic conditions to give carboxylic acid **14**. In the presence of BBr_3_, the perdemethylation of **14** and the concomitant lactonization gave urolithin C (**3**).^[^ ^5^ ^]^

**Scheme 4:**
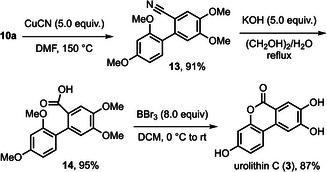
Synthetic route to Urolithin C (**2**).

Protosappanin A (**4**) contains medium‐sized cyclic ethers and has a notable bioactivity profile.^[^ ^23^, ^24^, ^25^ ^]^ Chu and Sun developed a three‐step ring‐expansion sequence to convert a 6–7–6‐tricyclic system to protosappanin A.^[^ ^26^, ^27^ ^]^ Aiming to synthesize **4**, our unified plan was to construct the central oxacycle from **5**. We conducted a phenol alkylation of **5** with methyl bromoacetate, introducing an ester group as a handle for the Parham cyclization^[^ ^28^, ^29^ ^]^ (Scheme 5). In the event, a metal‐halogen exchange of **15** with sec‐butyllithium generated aryl nucleophile **A**, which underwent a relatively underexplored 7‐exo‐trig cyclization^[^ ^30^ ^]^ to afford ketone **16** along with a small amount of hydrodebromination product, possibly resulting from a premature protonation of **A** by the carbonyl α‐proton. We have examined other alkyllithium reagents and lowered reaction temperature, but inferior yields of **16** were obtained in those trials. Since compound **16** is a known intermediate on the way to protosappanin A (**4**),^[^ ^26^, ^27^ ^]^ we achieved a formal total synthesis of **4** (Supporting Information).

**Scheme 5:**
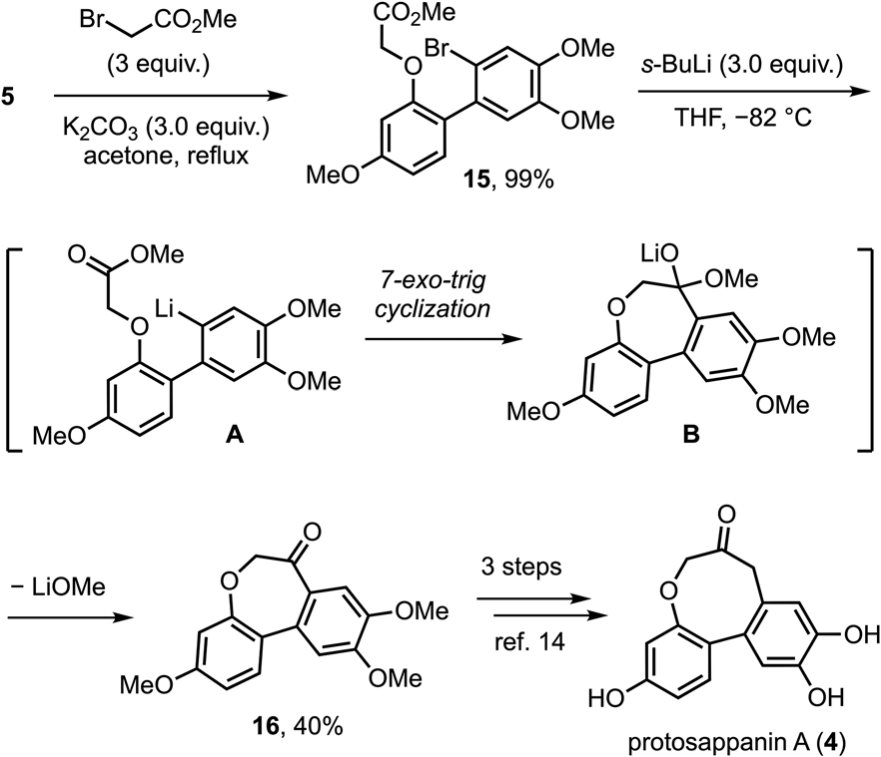
Synthetic route to and Protosappanin A (**4**).

In summary, we have developed a general approach to sappanin‐based natural products. The *α*‐arylation of CVE and the brominative aromatization were combined to assemble the privileged intermediate **5**, designed through the pattern‐recognition analysis, and subsequent strategic insertion of “spacers” to **5** or **10** enabled the total synthesis of caesappin A (**1**), caesappin B (**2**), urolithin C (**3**), and protosappanin A (**4**). We anticipate this approach will apply to accessing a broad range of dibenzo‐fused oxacyclic natural products.^[^ ^31^ ^]^

## Conflict of Interests

The authors declare no conflict of interest.

## Supporting information



Supporting Information

## Data Availability

The data that support the findings of this study are available in the supplementary material of this article.
