# Supplementary material for: Total Syntheses of Sappanin‐Based Natural Products Enabled by Pattern Recognition Analysis and Brominative Aromatization
Source: Chem Asian J. 2025 Oct 16;20(23):e00886. doi: 10.1002/asia.202500886 (PMC12696778; doi:10.1002/asia.202500886)
Supplement: Supplementary file 1 — Supporting Information [file ASIA-20-e00886-s001.pdf]

Total Syntheses of Sappanin-based Natural Products Enabled by Pattern Recognition Analysis and Brominative Aromatization

Wei-Ting Zhao,[a] Jing-Yu Liang,[a] Yen-Ku Wu*[a,b]

[a] *Department of Applied Chemistry and Center for Emergent Functional Matter Science, National Yang Ming Chiao Tung University, 1001 University Road, Hsinchu 30010, Taiwan*

[b] *Department of Chemistry, Chung Yuan Christian University, 200 Zhongbei Road, Taoyuan City 320314, Taiwan*

Supporting Information

Table of Contents

General Information……………………………………………………………… S-1

Synthesis and Characterization Data……………………..……………S-2−S-14

Spectral Comparison of 1, 2, 3, and 16………..……………………..…S-14−S-19

NMR Spectra…………………………………………………………S-20−S-51

General Information

All air-sensitive reactions were carried out with flame-dried glassware under N_2 atmosphere with the Schlenk line technique. Toluene was purified via a commercial solvent purification system. All other solvents (ACS grade) and commercially obtained reagents were used as received. Dimethylformamide (DMF) was dried by 4 Å molecular sieves and stored under N_2 atmosphere. Hexamethyldisilazane (HMDS) was freshly distilled over potassium hydroxide. Lithium hexamethyldisilazide (LiHMDS) was prepared prior to use by deprotonating HMDS in THF with *n*-BuLi (1.6 M in hexanes) at 0 ºC for 30 min. Reactions were monitored by thin-layer chromatography (TLC) on Merck silica gel 60 Å F254 plates and visualized via 254nm UV and $KMnO_4$ as color development agents. Reaction residues were purified via freshly prepared chromatography with silica gel (230-400 mash). Melting points were uncorrected. ^{1}H NMR (400 or 600 MHz) and ^{13}C NMR (125 MHz or 150 MHz) spectra were recorded at 25 °C in $CDCl_3$. Chemical shifts are reported in ppm, and coupling constants J are reported in Hz. High-resolution mass spectroscopy (HRMS) was performed on a TOF instrument with ESI in positive ionization mode. Infrared (IR) spectra were measured with ATR sampling system and were reported as wavenumber (cm^{-1}). High-resolution mass spectroscopy (HRMS) was performed on a TOF instrument with ESI or EI in positive ionization mode.

Synthesis and Characterization Data

3',4,4'-Trimethoxy-5,6-dihydro-[1,1'-biphenyl]-2(1H)-one (6)

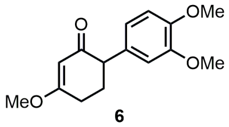

6

To a solution of hexamethyldisilazane (3.7 mL, 17.3 mmol, 2.2 equiv.) in toluene (20 mL) was slowly added a solution of *n*-butyl lithium (2.5 M in hexane; 6.4 mL, 16.0 mmol, 2.0 equiv.) at 0 °C under N_2 atmosphere. The resulting mixture was allowed to stir at the same temperature for 30 min. In the meantime, 3-methoxycyclohex-2-en-1-one (1.0 g, 7.94 mmol), 4-bromoveratrole (2.24 g, 10.3 mmol, 1.3 equiv.), $Pd(dba)_2$ (137 mg, 0.24 mmol, 3.0 mol%) and TTBP・HBF_4 (103 mg, 0.36 mmol, 4.5 mol%), toluene (60 mL) were sequentially introduced to a flask at room temperature, and the resulting solution was purged with N_2 for 2 min. The prepared LiHMDS solution was added dropwise to the flask containing the aryl bromide and the Pd catalyst at room temperature. After stirring for an additional 2 hours, saturated aqueous ammonium chloride was added to quench the reaction. The resulting mixture was extracted with ethyl acetate (100 mL x2), and the combined organic layers were washed with brine (100 mL), dried over anhydrous $MgSO_4$, filtered, and concentrated under reduced pressure. The crude product was purified by flash column chromatography (hexane/EA= 4:1 to 1:1) to afford **6** (1.91 g, 92%) as a yellow oil. R_f: 0.25 (hexane/EA = 1:1). **^{1}H NMR** (400 MHz, $CDCl_3$): δ 6.80 (d, J = 8.1 Hz, 1H), 6.72 − 6.68 (m, 1H), 6.67 (d, J = 2.1 Hz, 1H), 5.49 (s, 1H), 3.83 (s, 6H), 3.70 (s, 3H), 3.45 (dd, J = 9.7, 5.5 Hz, 1H), 2.61 − 2.38 (m, 2H), 2.30 − 2.13 (m, 2H); **^{13}C NMR** (100 MHz, $CDCl_3$): δ199.1, 177.8, 148.7, 147.8, 132.2, 120.1, 111.6, 111.1, 102.5, 55.7, 55.6, 55.6, 51.4, 29.3, 27.9. **IR** (film): 2936, 1652, 1605, 1514, 1379, 1248, 1190, 1026, 835 cm^{-1}; **HRMS** (EI) *m/z*: $[M]^+$ calcd. for $C_{15}H_{18}O_4$ 262.1199, found: 262.1190.

2'-Bromo-4,4',5'-trimethoxy-[1,1'-biphenyl]-2-ol (5)

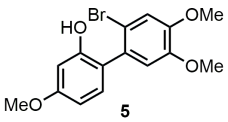

5

To a solution of **6** (450 mg, 1.71 mmol) in DCM (17 mL) was added I_2 (43 mg, 0.17 mmol, 10 mol%) in the dark. Subsequently, NBS (611 mg, 3.44 mmol, 2.0 equiv) was added into the reaction flask in four equal portions (Note: each portion was added at 15-minute intervals). After stirring for an additional 1 hour, 1 M $NaOH_{(aq)}$ was added to quench the reaction. The resulting mixture was extracted with DCM (50 mL x2), and the combined organic layers were washed with brine (50 mL), dried over anhydrous $MgSO_4$, filtered, and concentrated under reduced pressure. The residue (compound **7**) was dissolved in toluene (17 mL). To the solution was added 1,8-diazabicyclo[5.4.0]undec-7-ene (DBU; 261 mg, 3.44 mmol, 2.0 equiv.). After stirring for an additional 24 hours, 1 M $HCl_{(aq)}$ was added to quench the reaction. The resulting mixture was extracted with ethyl acetate (50 mL x2), and the combined organic layers were washed with brine (50 mL), dried over anhydrous $MgSO_4$, filtered, and concentrated under reduced pressure. The crude product was purified by flash column chromatography (hexane/EA= 10:1 to 2:1) to afford **5** (349 mg, 60% over 2 steps) as a pale-yellow solid (m.p. 145-147°C). R_f: 0.1 (hexane/EA = 4:1). **^{1}H NMR** (400 MHz, $CDCl_3$): δ 7.15 (s, 1H), 7.08 − 7.02 (m, 1H), 6.81 (s, 1H), 6.60 − 6.52 (m, 2H), 4.83 (brs, 1H), 3.92 (s, 3H), 3.85 (s, 3H), 3.83 (s, 3H) ; **^{13}C NMR** (100 MHz, $CDCl_3$): δ 160.9, 153.5, 149.5, 148.7, 131.1, 128.9, 120.2, 115.7, 114.8, 114.5, 106.6, 101.0, 56.2, 56.1, 55.3. **IR** (cast): 3456, 2937, 1618, 1496, 1206, 1170, 1032, 859, 784 cm^{-1}; **HRMS** (EI) *m/z*: $[M]^+$ calcd. for $C_{15}H_{15}O_4Br$ 338.0148, found: 338.0140.

2',3-Dibromo-4-ethoxy-4',5'-dimethoxy-5,6-dihydro-[1,1'-biphenyl]-2(1H)-one (9)

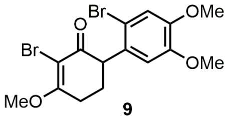

9

^{1}H NMR (400 MHz, $CDCl_3$): δ 7.02 (s, 1H), 6.58 (s, 1H), 4.08 (dd, *J* = 11.8, 5.1 Hz, 1H), 3.98 (s, 3H), 3.85 (s, 3H), 3.80 (s, 3H), 2.88 – 2.74 (m, 2H), 2.39 – 2.14 (m, 2H); **^{13}C NMR** (100 MHz, $CDCl_3$): δ 189.99, 172.30, 148.67, 148.47, 130.63, 115.50, 114.90, 111.97, 103.42, 77.31, 77.00, 76.68, 56.42, 56.11, 56.09, 51.53, 27.80, 26.35. **IR** (film): 2934, 1661, 1579, 1508, 1258, 1218, 1082, 909, 730 cm^{-1}; **HRMS** (EI) *m/z*: [M-H] calcd. for $C_{15}H_{15}O_4Br_2$ 417.9415, found: 416.9343.

2-Bromo-2',4,4',5-tetramethoxy-1,1'-biphenyl (10a)

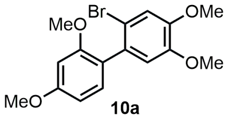

10a

To a solution of **5** (439 mg, 1.29 mmol) in acetone (13 mL) was added K_2CO_3 (718 mg, 5.2 mmol, 4.0 equiv.) and MeI (736 mg, 5.2 mmol, 4.0 equiv.). The reaction was refluxed at 70 °C overnight. After completion of the reaction, as indicated by TLC analysis, the reaction was cooled to room temperature, filtered, and then concentrated under reduced pressure. The crude product was purified by flash column chromatography (hexane/EA= 19:1 to 4:1) to afford **10a** (453 mg, 99%) as a colorless oil. **^{1}H NMR** (400 MHz, $CDCl_3$): δ 7.11 (s, 1H), 7.11 – 7.07 (m, 1H), 6.79 (s, 1H), 6.58 – 6.54 (m, 2H), 3.90 (s, 3H), 3.86 (s, 3H), 3.84 (s, 3H), 3.78 (s, 3H); **^{13}C NMR** (100 MHz, $CDCl_3$): δ 160.6, 157.6, 148.5, 147.9, 131.5, 123.0, 115.2, 114.6, 114.5, 104.0, 98.7, 56.1, 55.9, 55.6, 55.3. **IR** (film): 2935, 1610, 1495, 1413, 1250, 1207, 1032, 783cm^{-1}; **HRMS** (EI) *m/z*: $[M]^+$ calcd. for $C_{16}H_{17}O_4Br$ 352.0305, found: 352.0304.

2-Allyl-2',4,4',5-tetramethoxy-1,1'-biphenyl (11a)

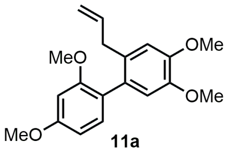

11a

To a solution of **10a** (400 mg, 1.13 mmol) in DMF (12 mL) was added $Pd(PPh_3)_4$ (132 mg, 0.113 mmol, 10 mol%) and allyltributyltin (748 mg, 2.26 mmol, 2.0 equiv.). The reaction was stirred at 130 °C for 30 hours. Saturated aqueous potassium fluoride was added to quench the reaction. The resulting mixture was extracted with ethyl acetate (50 mL x2), and the combined organic layers were sequentially washed with saturated aqueous potassium fluoride (25 mL), DI water (25 mL), and brine (25 mL), dried over anhydrous $MgSO_4$, filtered, and concentrated under reduced pressure. The crude product was purified by flash column chromatography (hexane/EA= 10:1 to 2:1) to afford **11a** (325 mg, 89%) as a colorless oil. R_f: 0.3 (hexane/EA = 4:1). **^{1}H NMR** (400 MHz, $CDCl_3$): δ 7.10 – 7.05 (m, 1H), 6.78 (s, 1H), 6.71 (s, 1H), 6.57 – 6.52 (m, 2H), 5.96 – 5.72 (m, 1H), 5.07 – 4.85 (m, 2H), 3.90 (s, 3H), 3.86 (s, 3H), 3.85 (s, 3H), 3.75 (s, 3H), 3.25 – 3.08 (m, 2H); **^{13}C NMR** (100 MHz, $CDCl_3$): δ 160.1, 157.6, 147.9, 146.7, 137.9, 131.7, 130.9, 129.9, 122.8, 115.1, 113.7, 111.8, 103.9, 98.4, 55.8, 55.7, 55.3, 55.3, 37.3. **IR** (film): 2953, 1608, 1498, 1463, 1252, 1207, 1158, 1048, 833 cm^{-1}; **HRMS** (EI) *m/z*: $[M]^+$ calcd. for $C_{19}H_{22}O_4$ 314.1513, found: 314.1521.

1-(2',4,4',5-Tetramethoxy-[1,1'-biphenyl]-2-yl)propan-2-one (12a)

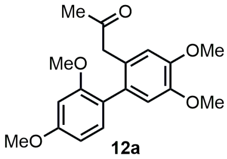

12a

To a solution of **11a** (300 mg, 0.955 mmol) in DMF (5 mL) and H_2O (1 mL) was added $PdCl_2$ (34 mg, 0.191 mmol, 20 mol%) and CuCl (95 mg, 0.955 mmol, 1.0 equiv.); air

was continuously bubbled into the reaction solution over 24 hours. The resulting mixture was diluted with DI water and extracted with ethyl acetate (50 mL x3), and the combined organic layers were washed with brine (50 mL), dried over anhydrous $MgSO_4$, filtered, and concentrated under reduced pressure. The crude product was purified by flash column chromatography (hexane/EA= 10:1 to 1:1) to afford **12a** (230 mg, 73%) as a colorless oil. R*f*: 0.15 (hexane/EA = 2:1). **^{1}H NMR** (400 MHz, $CDCl_3$): δ 7.08 – 6.99 (m, 1H), 6.73 (s, 1H), 6.71 (s, 1H), 6.56 – 6.48 (m, 2H), 3.88 (s, 3H), 3.83 (s, 3H), 3.83 (s, 3H), 3.69 (s, 3H), 3.45 (s, 2H), 1.95 (s, 3H); **^{13}C NMR** (100 MHz, $CDCl_3$): δ 207.1, 160.3, 157.3, 147.9, 147.4, 131.7, 130.6, 125.7, 122.2, 113.8, 112.6, 104.2, 98.3, 55.7, 55.7, 55.2, 55.1, 48.0, 29.0. **IR** (film): 2935, 1706, 1606, 1499, 1300, 1208, 1157, 832 cm^{-1}; **HRMS** (EI) *m/z*: $[M]^+$ calcd. for $C_{19}H_{22}O_5$ 330.1462, found: 330.1453.

2',4,4',5-Tetrakis(benzyloxy)-2-bromo-1,1'-biphenyl (10b)

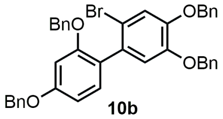

10b

To a solution of **5** (250 mg, 0.737 mmol) in DCM (7 mL) was slowly added a solution of BBr_3 (1.0 M in DCM, 6.0 mL, 6.0 mmol, 8.0 equiv.) at 0 °C under N_2 atmosphere over 5 minutes. The resulting mixture was stirred at room temperature for 24 hours. MeOH was added to quench the reaction at 0 °C. The mixture was concentrated under reduced pressure, and the residue was diluted with ethyl acetate. The organic layer was sequentially washed with DI water and brine, dried over anhydrous $MgSO_4$, filtered, and concentrated under reduced pressure. The crude product was dissolved in acetone (8 mL). To it was added K_2CO_3 (814 mg, 5.91 mmol) and BnBr (0.53 mL, 4.42 mmol) at room temperature. The reaction was stirred at 70 °C overnight. After completion of

the reaction, as indicated by TLC analysis, the reaction was cooled to room temperature, filtered, and concentrated under reduced pressure. The crude product was purified by flash column chromatography (hexane/EA= 99:1 to 19:1) to afford **10b** (436 mg, 90% over 2 steps) as a colorless oil. **^{1}H NMR** (400 MHz, $CDCl_3$): δ 7.55 − 7.23 (m, 21H), 7.12 (d, J = 8.2 Hz, 1H), 6.97 (s, 1H), 6.73 − 6.62 (m, 2H), 5.17 (s, 2H), 5.08 (s, 4H), 5.03 (s, 2H); **^{13}C NMR** (100 MHz, $CDCl_3$): δ 159.6, 156.7, 148.5, 147.8, 137.0, 136.9, 136.8, 136.7, 132.3, 131.6, 128.6, 128.5, 128.4, 128.3, 128.0, 127.9, 127.8, 127.6, 127.5, 127.3, 126.8, 123.6, 118.6, 118.3, 115.3, 105.6, 101.2, 71.4, 71.3, 70.3, 70.1. **IR** (film): 3030, 2915, 1606, 1489, 1247, 1169, 1024, 734 cm^{-1}; **HRMS** (EI) *m/z*: $[M]^+$ calcd. for $C_{40}H_{33}O_4Br$ 656.1557, found: 656.1600.

2-allyl-2',4,4',5-Tetrakis(benzyloxy)-1,1'-biphenyl (11b)

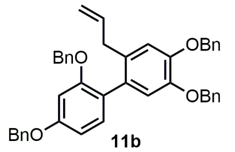

11b

To a solution of **10b** (140 mg, 0.216 mmol) in DMF (5 mL) was added $Pd(PPh_3)_4$ (25 mg, 0.022 mmol, 10 mol%) and allyltributyltin (143 mg, 0.432 mmol, 2.0 equiv.). The reaction was stirred at 130 °C for 30 hours. Saturated aqueous potassium fluoride was added to quench the reaction. The resulting mixture was extracted with ethyl acetate (25 mL x2). The combined organic layers were sequentially washed with saturated aqueous potassium fluoride (25 mL), DI water (25 mL), and brine (25 mL), dried over anhydrous $MgSO_4$, filtered, and concentrated under reduced pressure. The crude product was purified by flash column chromatography (hexane/EA= 99:1 to 19:1) to afford **11b** (132 mg, 80%) as a colorless oil. R*f*: 0.65 (hexane/EA = 10:1). **^{1}H NMR** (400 MHz, $CDCl_3$): δ 7.53 − 7.24 (m, 18H), 7.19 (dd, J = 7.9, 1.9 Hz, 2H), 7.06 (d, J = 7.9 Hz, 1H), 6.89 (d, J = 9.0 Hz, 2H), 6.70 − 6.59 (m, 2H), 5.78 (m, 1H), 5.19 (s, 2H),

5.10 (s, 2H), 5.07 (s, 2H), 4.97 (s, 2H), 4.95 − 4.82 (m, 2H), 3.17 (s, 2H); ^{13}C **NMR** (100 MHz, $CDCl_3$): δ 159.2, 156.7, 148.0, 146.8, 137.8, 137.5, 137.5, 137.1, 136.9, 132.0, 131.8, 131.0, 128.6, 128.4, 128.3, 128.3, 128.3, 128.0, 127.6, 127.6, 127.5, 127.4, 127.3, 126.6, 123.7, 117.8, 115.9, 115.3, 105.8, 101.4, 37.3. **IR** (film): 2953, 2869, 1604, 1496, 1376, 1169, 1025, 733, 694 cm^{-1}; **HRMS** (EI) *m/z*: $[M]^+$ calcd. for $C_{43}H_{38}O_4$ 618.2765, found: 618.2770.

1-(2',4,4',5-Tetrakis(benzyloxy)-[1,1'-biphenyl]-2-yl)propan-2-one (12b)

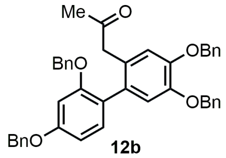

To a solution of **11b** (52 mg, 0.085 mmol, 1.0 equiv) in DMF (4 mL) and H_2O (1 mL) was added $PdCl_2$ (3.0 mg, 0.017 mmol, 20 mol%) and CuCl (8.4 mg, 0.085 mmol, 1.0 equiv); ; air was continuously bubbled into the reaction solution over 24 hours. The resulting mixture was diluted with DI water and extracted with ethyl acetate (25 mL x3). The combined organic layers were washed with brine (25 mL), dried over anhydrous $MgSO_4$, filtered, and concentrated under reduced pressure. The crude product was purified by flash column chromatography (hexane/EA= 99:1 to 9:1) to afford **12b** (37 mg, 69%) as a colorless oil. R_f: 0.3 (hexane/EA = 9:1). ^{1}H **NMR** (400 MHz, $CDCl_3$): δ 7.49 − 7.26 (m, 17H), 7.26 − 7.23 (m, 1H), 7.18 − 7.13 (m, 2H), 7.00 (d, J = 8.2 Hz, 1H), 6.87 (s, 1H), 6.82 (s, 1H), 6.70 − 6.58 (m, 2H), 5.18 (s, 2H), 5.11 (s, 2H), 5.06 (s, 2H), 4.94 (s, 2H), 3.51 − 3.38 (m, 2H), 1.80 (s, 3H); ^{13}C **NMR** (100 MHz, $CDCl_3$): δ 207.4, 159.4, 156.5, 148.0, 147.6, 137.3, 136.8, 136.7, 131.8, 131.7, 128.6, 128.4, 128.4, 128.3, 128.0, 127.7, 127.7, 127.6, 127.5, 127.4, 127.3, 126.8, 126.7, 123.2, 117.7, 116.5, 106.1, 101.5, 71.3, 70.2, 70.2, 48.3, 29.1. **IR** (film): 3030, 2866, 1706, 1604, 1497, 1453, 1169, 1026, 735 cm^{-1}; **HRMS** (EI) *m/z*: [M]+ calcd. for

$C_{43}H_{38}O_5$ 634.2714, found: 634.2710.

Caesappin B (2)

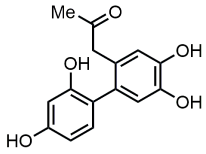

caesappin B (**2**)

To a solution of **12b** (37 mg, 0.0581 mmol) in ethyl acetate (5 mL) was added Pd/C (8 mg, 10 wt.%), and $H_{2(g)}$ was continuously bubbled into the reaction solution. After completion of the reaction, as indicated by TLC analysis, the reaction was filtered and concentrated under reduced pressure. The crude product was purified by HPLC (LaboACE LC-5060; solvent: ethyl acetate; rate:10 mL/min) to afford **2** (15 mg, 90%) as a colorless solid. **^{1}H NMR** (400 MHz, DMSO-d_6): δ 9.17 (s, 1H), 9.02 (s, 1H), 8.72 (s, 1H), 8.70 (s, 1H), 6.66 (d, J = 8.2 Hz, 1H), 6.55 (s, 1H), 6.47 (s, 1H), 6.32 (d, J = 2.4 Hz, 1H), 6.20 (dd, J = 8.2, 2.4 Hz, 1H), 3.31 (s, 2H), 1.83 (s, 3H); **^{13}C NMR** (100 MHz, DMSO-d_6): δ 206.8, 157.3, 155.0, 143.8, 143.4, 131.5, 130.1,124.7, 118.9, 117.9, 117.1, 106.2, 102.3, 47.3, 29.1.

Caesappin A (1)

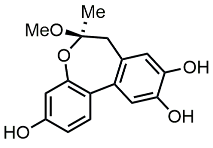

caesappin A (**1**)

To a solution of **2** (15 mg, 0.0547 mmol) in MeOH (5 mL) was added *p*-TsOH (10 mg, 0.0547 mmol) and trimethyl orthoformate (1.0 mL). The resulting mixture was stirred at room temperature for 24 hours. After completion of the reaction, as indicated by TLC analysis, the reaction was concentrated under reduced pressure. The crude product was

purified by HPLC (LaboACE LC-5060; solvent: ethyl acetate; rate:10 mL/min) to afford **1** (14 mg, 85%) as a colorless solid. **^{1}H NMR** (400 MHz, DMSO-d_6): δ 9.48 (s, 1H), 8.86 (s, 1H), 8.86 (s, 1H), 7.11 (d, J = 8.3 Hz, 1H), 6.74 (s, 1H), 6.67 (s, 1H), 6.62 (dd, J = 8.3, 2.4 Hz, 1H), 6.43 (d, J = 2.4 Hz, 1H), 3.42 (s, 3H), 2.54 (d, J = 13.9 Hz, 1H), 2.44 (d, J = 13.9 Hz, 1H), 1.36 (s, 3H); **^{13}C NMR** (100 MHz, DMSO-d_6): δ 157.08, 152.5, 144.4, 144.0, 128.7, 128.4, 125.5, 125.3, 116.8, 114.4, 111.9, 111.6, 110.2, 48.9, 42.6, 21.0.

2',4,4',5-Tetramethoxy-[1,1'-biphenyl]-2-carbonitrile (13)

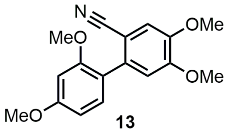

13

To a solution of **10a** (93 mg, 0.31 mmol) in DMF (0.9 mL) was added CuCN (119 mg, 1.55 mmol, 1.0 equiv.). The reaction was stirred at 150 °C for 24 hours. The resulting mixture was diluted with DI water and extracted with ether (10 mL x3). The combined organic layers were washed with brine (10 mL), dried over anhydrous $MgSO_4$, filtered, and concentrated under reduced pressure. The crude product was purified by flash column chromatography (hexane/EA= 19:1 to 4:1) to afford **13** (72 mg, 91%) as a colorless solid (m.p. 131-133°C). R*f*: 0.2 (hexane/EA = 4:1). **^{1}H NMR** (400 MHz, $CDCl_3$): δ 7.22 – 7.18 (m, 1H), 7.12 (s, 1H), 6.88 (s, 1H), 6.60 – 6.55 (m, 2H), 3.92 (s, 3H), 3.92 (s, 3H), 3.86 (s, 3H), 3.82 (s, 3H); **^{13}C NMR** (100 MHz, $CDCl_3$): δ 161.3, 157.5, 152.1, 147.7, 136.8, 131.4, 119.9, 119.1, 114.3, 113.6, 104.7, 104.5, 99.0, 56.1, 56.0, 55.5, 55.4. **IR** (cast): 3007, 2934, 2842, 2218, 1499, 1206, 1159, 752 cm^{-1}; **HRMS** (EI) *m/z*: $[M]^+$ calcd. for $C_{17}H_{17}NO_4$ 299.1158, found: 299.1152.

2',4,4',5-Tetramethoxy-[1,1'-biphenyl]-2-carboxylic acid (14)

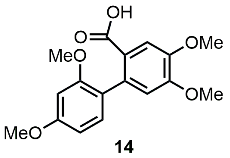

14

To a solution of **13**(58 mg, 0.19 mmol) in ethylene glycol (1.0 mL) and H_2O (0.5 mL) was added KOH (200 mg, 3.77 mmol, 20.0 equiv.). The reaction was refluxed overnight. After completion of the reaction, as indicated by TLC analysis. 1 M $HCl_{(aq)}$ was added to quench the reaction. The resulting mixture was extracted with ethyl acetate (25 mL x3), and the combined organic layers were washed with brine (25 mL), dried over anhydrous $MgSO_4$, filtered, and concentrated under reduced pressure. The crude product was purified by flash column chromatography (hexane/EA= 2:1 to 0:1) to afford **14** (59 mg, 95%) as a colorless solid (m.p. 227-228℃). R_f: 0.3 (EA). **^{1}H NMR** (400 MHz, $CDCl_3$): δ 7.52 (s, 1H), 7.15 (d, J = 8.3 Hz, 1H), 6.74 (s, 1H), 6.56 (dd, J = 8.3, 2.4 Hz, 1H), 6.49 (d, J = 2.4 Hz, 1H), 3.95 (s, 3H), 3.91 (s, 3H), 3.86 (s, 3H), 3.71 (s, 3H); **^{13}C NMR** (100 MHz, $CDCl_3$): δ 171.7, 160.5, 157.3, 152.0, 147.4, 133.4, 130.1, 123.1, 121.9, 114.3, 113.0, 104.2, 98.6, 56.0, 56.0, 55.3, 55.2. **IR** (cast): 2936, 2358, 1733, 1684, 1502, 1259, 1207, 1045, 755 cm^{-1}; **HRMS** (EI) *m/z*: $[M]^+$ calcd. for $C_{17}H_{18}O_6$ 318.1103, found: 318.1098.

Urolithin C (3)

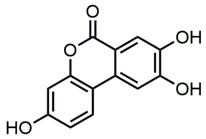

urolithin C (**3**)

To a solution of **14** (60 mg, 0.19 mmol) in DCM (1.9 mL) was slowly added a solution of BBr_3 (1.0 M in DCM, 1.9 mL, 1.9 mmol, 10.0 equiv.) at 0 °C under N_2 atmosphere. After completion of the reaction, as indicated by TLC analysis, saturated $NaHCO_{3(aq)}$

was added to quench the reaction at 0°C. The mixture was diluted with DCM, and the organic layer was sequentially washed with DI water and brine, dried over anhydrous $MgSO_4$, filtered, and concentrated under reduced pressure. The crude product was purified by flash column chromatography (EA) to afford **3** (40 mg, 87%) as a colorless solid. R_f: 0.1 (EA). **^{1}H NMR** (400 MHz, DMSO-d_6): δ 10.13 (brs, 3H), 7.84 (d, J = 8.7 Hz, 1H), 7.49 (s, 1H), 7.43 (s, 1H), 6.79 (dd, J = 8.7, 2.4 Hz, 1H), 6.69 (d, J = 2.4 Hz, 1H); **^{13}C NMR** (100 MHz, DMSO-d_6): δ 160.3, 158.6, 153.4, 151.4, 146.1, 129.2, 123.7, 114.2, 112.8, 110.9, 109.8, 106.8, 102.8.

Methyl 2-((2'-bromo-4,4',5'-trimethoxy-[1,1'-biphenyl]-2-yl)oxy)acetate (15)

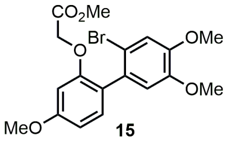

15

To a solution of **5** (620 mg, 1.83 mmol) in acetone (20 mL) was added K_2CO_3 (760 mg, 5.5 mmol, 3.0 equiv.) and methyl bromoacetate (0.52 mg, 5.5 mmol, 3.0 equiv.). The reaction was stirred at 70 °C for 12 hours. After completion of the reaction, as indicated by TLC analysis, the reaction was cooled to room temperature, filtered, and concentrated under reduced pressure. The crude product was purified by flash column chromatography (hexane/EA= 5:1 to 2:1) to afford **15** (745 mg, 99%) as a yellow oil. R_f: 0.2 (hexane/EA = 4:1). **^{1}H NMR** (400 MHz, $CDCl_3$): 7.16 (d, J = 8.4 Hz, 1H), 7.11 (s, 1H), 6.91 (s, 1H), 6.61 (dd, J = 8.4, 2.4 Hz, 1H), 6.44 (d, J = 2.4 Hz, 1H), 4.58 (s, 2H), 3.90 (s, 3H), 3.85 (s, 3H), 3.83 (s, 3H), 3.75 (s, 3H); **^{13}C NMR** (100 MHz, $CDCl_3$): δ 169.2, 160.3, 155.9, 148.6, 147.9, 132.2, 130.8, 123.4, 115.2, 114.8, 114.2, 105.56 100.3, 65.9, 56.1, 55.9, 55.4, 52.1. **IR** (film): 3003, 2948, 2833, 1757, 1610, 1494, 1205, 1161, 1023, 784 cm^{-1}; **HRMS** (ESI) m/z: $[M+Na]^+$ calcd. for $C_{18}H_{19}NaBrO_6$ 410.0365,

found: 433.0257.

3,9,10-Trimethoxydibenzo[b,d]oxepin-7(6H)-one (16)

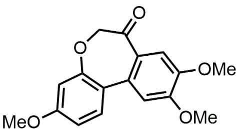

16

To a solution of **15** (100 mg, 0.25 mmol) in THF (2.5 mL) was slowly added a solution of *sec*-butyllithium (0.8 mL, 1.0 mmol, 1.3 M in cyclohexane/hexane) at −82 °C (an ethanol-liquid nitrogen bath) under N_2 atmosphere. The reaction mixture was stirred at −82 °C for 1 hour. After completion of the reaction, as indicated by TLC analysis, the mixture was quenched with DI water and allowed to warm to rt. It was extracted with ethyl acetate (25 mL x3). The combined organic layers were washed with brine (25 mL), dried over anhydrous $MgSO_4$, filtered, and concentrated under reduced pressure. The crude product was purified by flash column chromatography (hexane/EA/DCM= 12:1:1 to 6:1:1) to afford **16** (29 mg, 40%) as a colorless oil. R_f: 0.35 (hexane/EA/DCM= 4:1:1). **^{1}H NMR** (400 MHz, $CDCl_3$): δ 7.48 (s, 1H), 7.47 (d, J = 8.6 Hz, 1H), 6.92 (s, 1H), 6.85 (dd, J = 8.6, 2.7 Hz, 1H), 6.76 (d, J = 2.7 Hz, 1H), 4.76 (s, 2H), 4.00 (s, 3H), 3.97 (s, 3H), 3.85 (s, 3H); **^{13}C NMR** (100 MHz, $CDCl_3$): δ 201.5, 160.8, 157.8, 153.4, 148.2, 131.9, 130.5, 127.8, 125.0, 112.1, 111.5, 111.2, 106.1, 81.9, 56.1, 56.0, 55.5. **IR** (film): 3007, 2934, 2844, 1666, 1597, 1498, 1270, 1210, 1034 cm^{-1}; **HRMS** (ESI) *m/z*: $[M+H]^+$ calcd. for $C_{17}H_{17}O_5$ 300.0998, found: 301.1066.

Caesappin A: ^{1}H NMR Spectral Comparison

Liang's report (500 MHz, DMSO-d_6)[1]		Synthetic (400 MHz, DMSO-d_6)	
δH (ppm)	integration, multiplicity	δH (ppm)	integration, multiplicity
9.46	1H, s	9.48	1H, s
8.84	1H, s	8.86	1H, s
8.84	1H, s	8.86	1H, s
7.12	1H, d (8.3 Hz)	7.11	1H, d (8.3 Hz)
6.75	1H, s	6.74	1H, s
6.68	1H, s	6.67	1H, s
6.63	1H, dd (8.3, 2.0 Hz)	6.62	1H, dd (8.3, 2.4 Hz)
6.44	1H, d (2.0 Hz)	6.43	1H, d (2.4 Hz)
3.42	3H, s	3.42	3H, s
2.50	1H, s	2.54	1H, d (13.9 Hz)
2.50	1H, s	2.44	1H, d (13.9 Hz)
1.36	3H, s	1.36	3H, s

Caesappin A: ^{13}C NMR Spectral Comparison

Liang's report (125 MHz, DMSO-d_6)[1]	Synthetic (100 MHz, DMSO-d_6)
δC (ppm)	δC (ppm)
157.1	157.1
152.6	152.6
144.4	144.4
144.1	144.1
128.8	128.8
128.4	128.5

125.6	125.5
125.3	125.3
116.8	116.8
114.4	114.4
111.9	111.9
111.7	111.7
110.3	110.3
48.9	48.9
42.6	42.6
21.0	21.0

Caesappin B: ^{1}H NMR Spectral Comparison

Liang's report (500 MHz, DMSO-d_6)[1]		Synthetic (400 MHz, DMSO-d_6)	
δH (ppm)	integration, multiplicity	δH (ppm)	integration, multiplicity
9.17	1H, s	9.17	1H, s
9.01	1H, s	9.02	1H, s
8.72	1H, s	8.72	1H, s
8.69	1H, s	8.70	1H, s
6.66	1H, d (8.2 Hz)	6.66	1H, d (8.2 Hz)
6.55	1H, s	6.55	1H, s
6.47	1H, s	6.47	1H, s
6.31	1H, d (2.3 Hz)	6.32	1H, d (2.4 Hz)
6.21	1H, dd (8.3, 2.3 Hz)	6.20	1H, dd (8.2, 2.4 Hz)
3.34	1H, s	3.31	2H, s
3.34	1H, s		

1.83	3 H, s	1.83	3 H, s

Caesappin B: ^{13}C NMR Spectral Comparison

Liang's report (125 MHz, DMSO-d_6)[1] δC (ppm)	Synthetic (100 MHz, DMSO-d_6) δC (ppm)
206.9	206.9
157.3	157.3
155.0	155.0
143.8	143.8
143.4	143.4
131.5	131.6
130.1	130.1
124.8	124.7
119.0	119.0
118.0	118.0
117.2	117.2
106.2	106.2
102.4	102.4
47.3	47.3
29.1	29.1

Urolithin C: ^{1}H NMR Spectral Comparison

Thasana's report (400 MHz, DMSO-d$_6$)[2]		Synthetic (400 MHz, DMSO-d$_6$)	
δH (ppm)	integration, multiplicity	δH (ppm)	integration, multiplicity
10.12	3H, brs	10.13	3H, brs
7.85	1H, d (8.7 Hz)	7.84	1H, d (8.7 Hz)
7.47	1H, s	7.49	1H, s
7.41	1H, s	7.43	1H, s
6.77	1H, dd (8.2, 2.4Hz)	6.79	1H, dd (8.7, 2.4 Hz)
6.67	1H, d (2.4 Hz)	6.69	1H, d (2.4 Hz)

Urolithin C: ^{13}C NMR Spectral Comparison

Thasana's report (100 MHz, DMSO-d$_6$)[2]	Synthetic (100 MHz, DMSO-d$_6$)
δC (ppm)	δC (ppm)
160.7	160.3
159.0	158.6
153.8	153.5
151.8	151.5
146.5	146.2
129.6	129.2
124.1	124.7
114.6	114.2
113.2	112.8
111.3	110.9

110.2	109.8
107.2	106.8
103.4	102.8

Compound 16 (Chu &Sun's intermediate): ^{1}H NMR Spectral Comparison

Chu & Sun's report (400 MHz, $CDCl_3$)[3]		Synthetic (400 MHz, $CDCl_3$)	
δH (ppm)	integration, multiplicity	δH (ppm)	integration, multiplicity
7.49	1H, s	7.48	1H, s
7.47	1H, d (8.8 Hz)	7.47	1H, d (8.6 Hz)
6.93	1H, s	6.92	1H, s
6.86	1H, dd (8.6, 2.4 Hz)	6.85	1H, dd (8.6, 2.7 Hz)
6.76	1H, d (2.4 Hz)	6.76	1H, d (2.7 Hz)
4.77	2H, s	4.76	2H, s
4.00	3H, s	4.00	3H, s
3.97	3H, s	3.97	3H, s
3.86	3H, s	3.85	3H, s

Compound 16 (Chu &Sun's intermediate): ^{13}C NMR Spectral Comparison

Chu & Sun's report (400 MHz, $CDCl_3$)[3]	Synthetic (400 MHz, $CDCl_3$)
δC (ppm)	δC (ppm)
201.7	201.5

161.1	160.8
158.1	157.9
153.7	153.5
148.5	148.2
132.2	131.9
130.7	130.5
128.1	127.8
125.3	125.1
112.3	112.1
111.8	111.5
111.5	111.3
106.3	106.1
82.2	81.9
56.3	56.1
56.3	56.1
55.7	55.5

References:

1. Z. Wang, J.-B. Sun, W. Qu, F.-Q. Guan, L.-Z. Li, J.-Y. Liang, *Fitoterapia* **2014**, *92*, 280–284.

2. P. Nealmongkol, K. Tangdenpaisal, S. Sitthimonchai, S. Ruchirawat, N. Thasana, *Tetrahedron* **2013**, *69*, 9277–9283.

3. J. Liu, X. Zhou, C. Wang, W. Fu, W. Chu, Z. Sun, *Chem. Commun.* **2016**, *52*, 5152–5155.

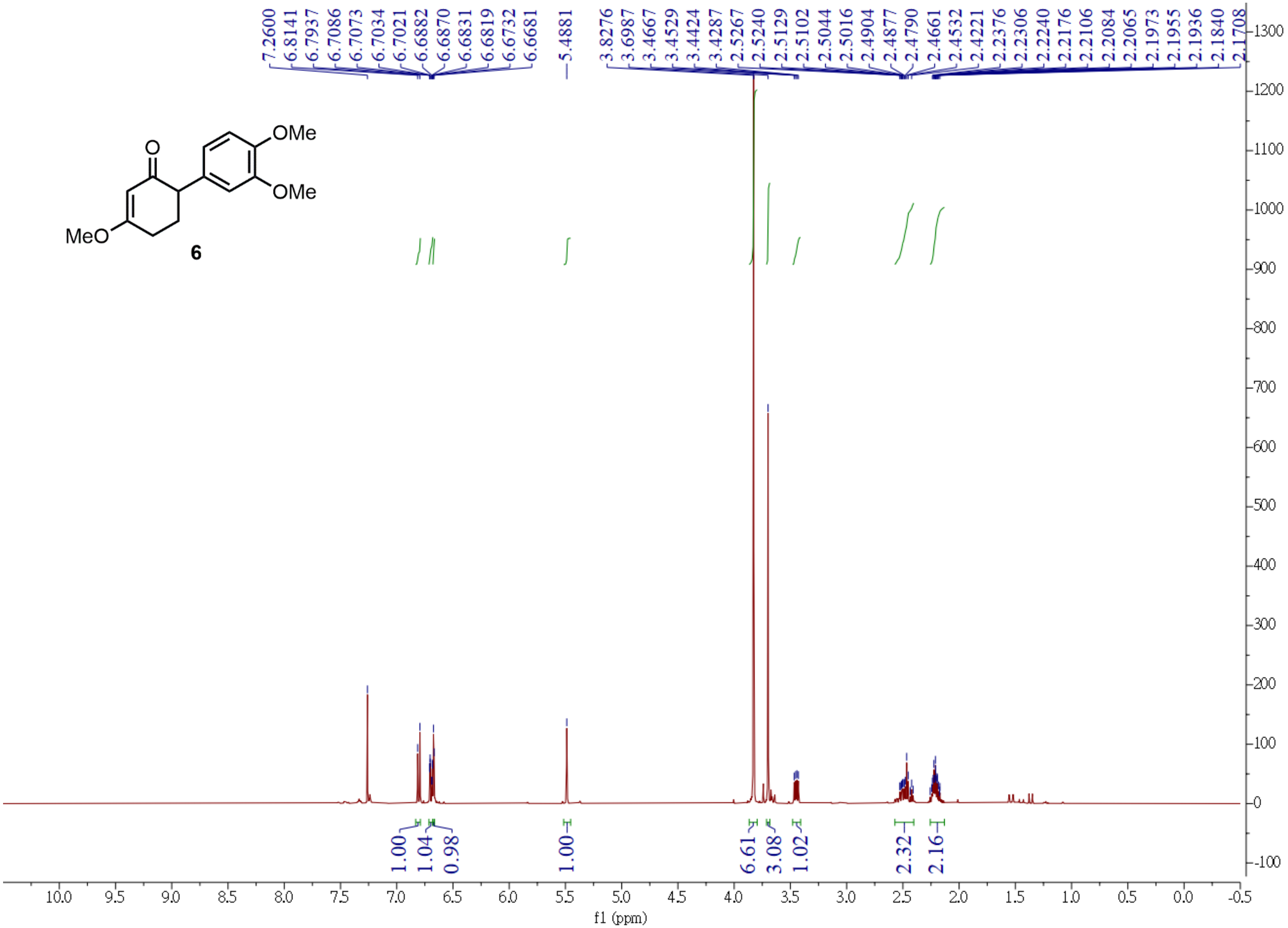

^{1}H NMR Spectrum of Compound 6 (CDCl$_3$, 400 MHz)

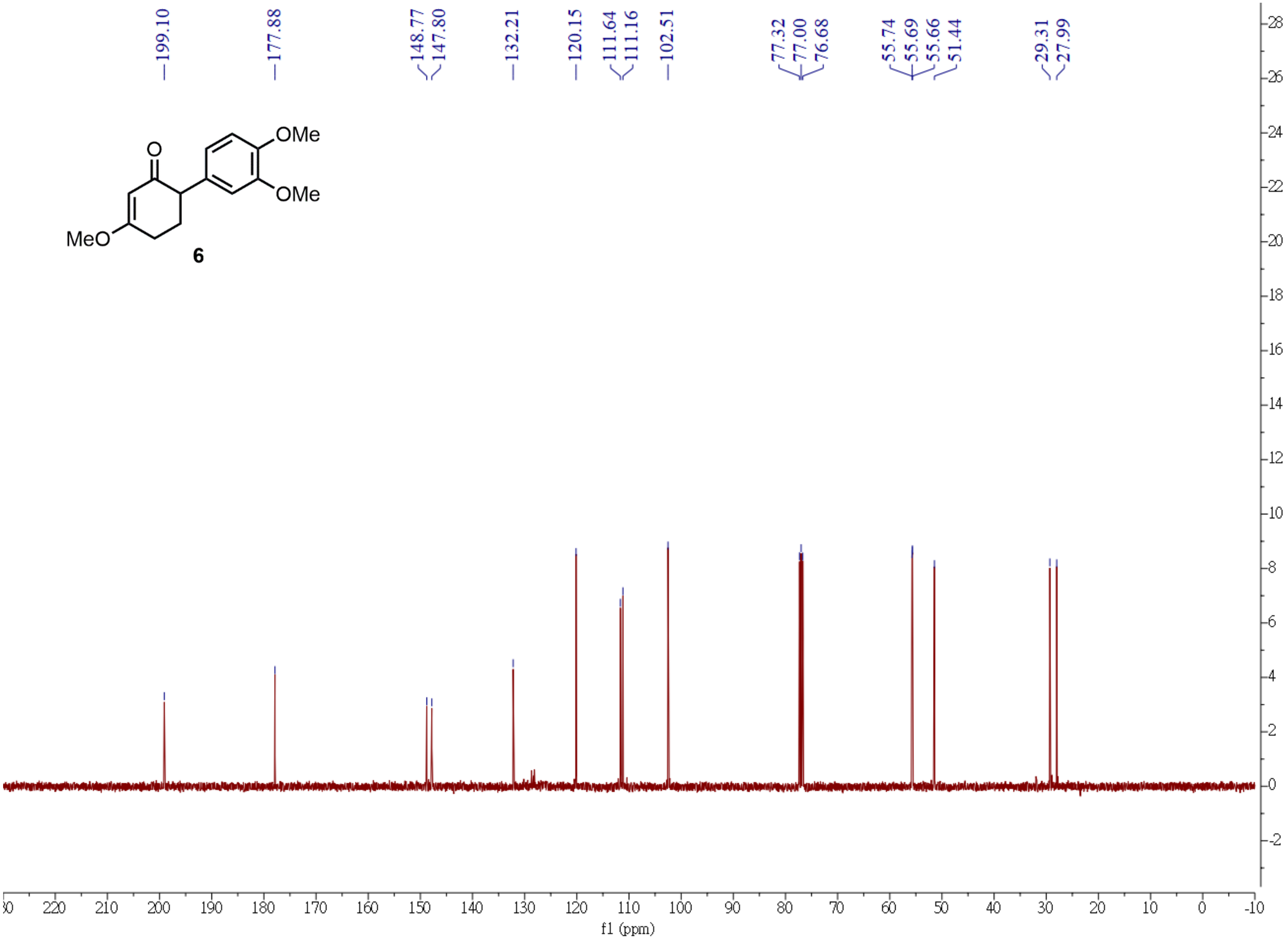

^{13}C NMR Spectrum of Compound 6 ($CDCl_3$, 100 MHz)

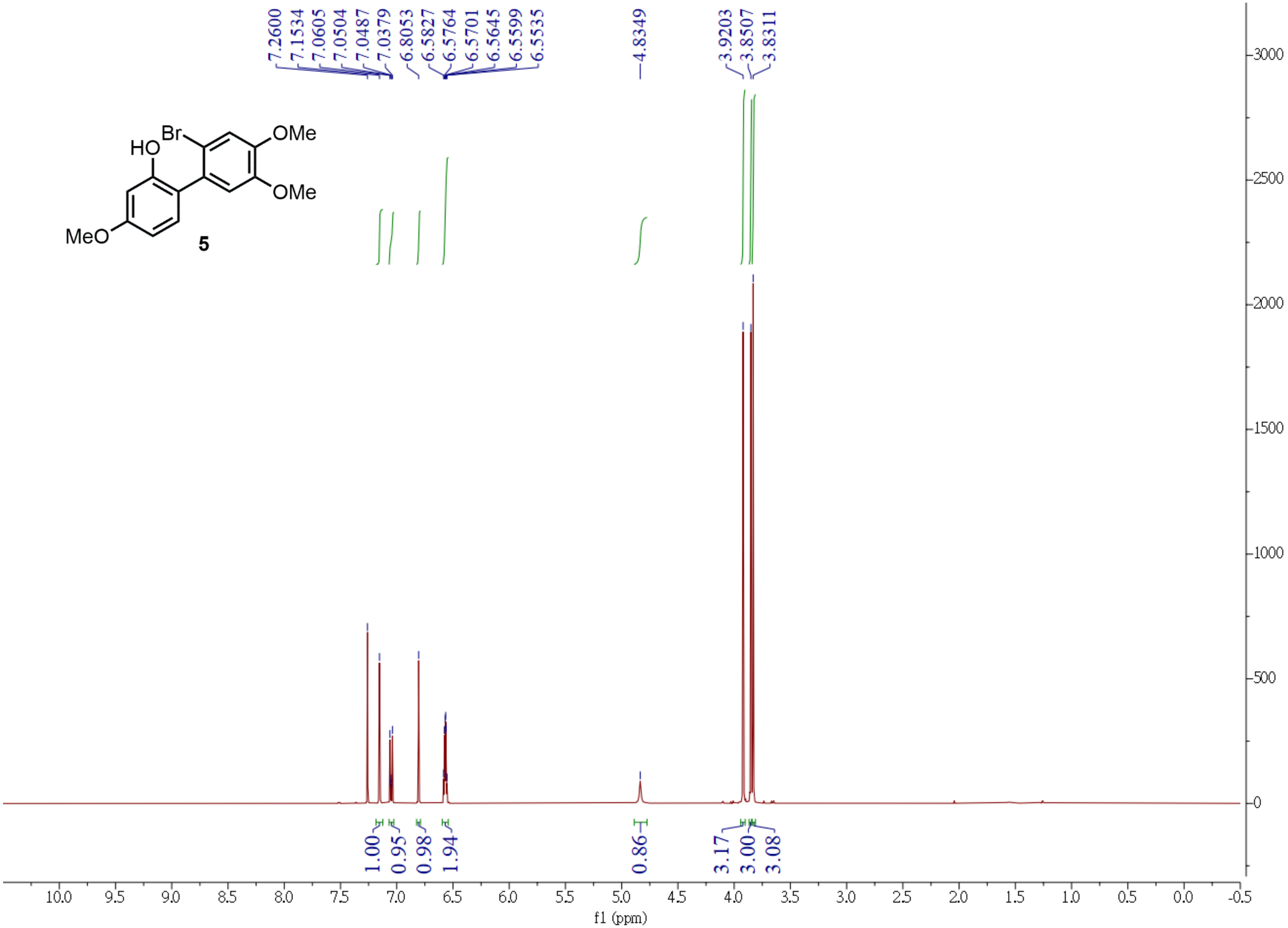

^{1}H NMR Spectrum of Compound 5 ($CDCl_3$, 400 MHz)

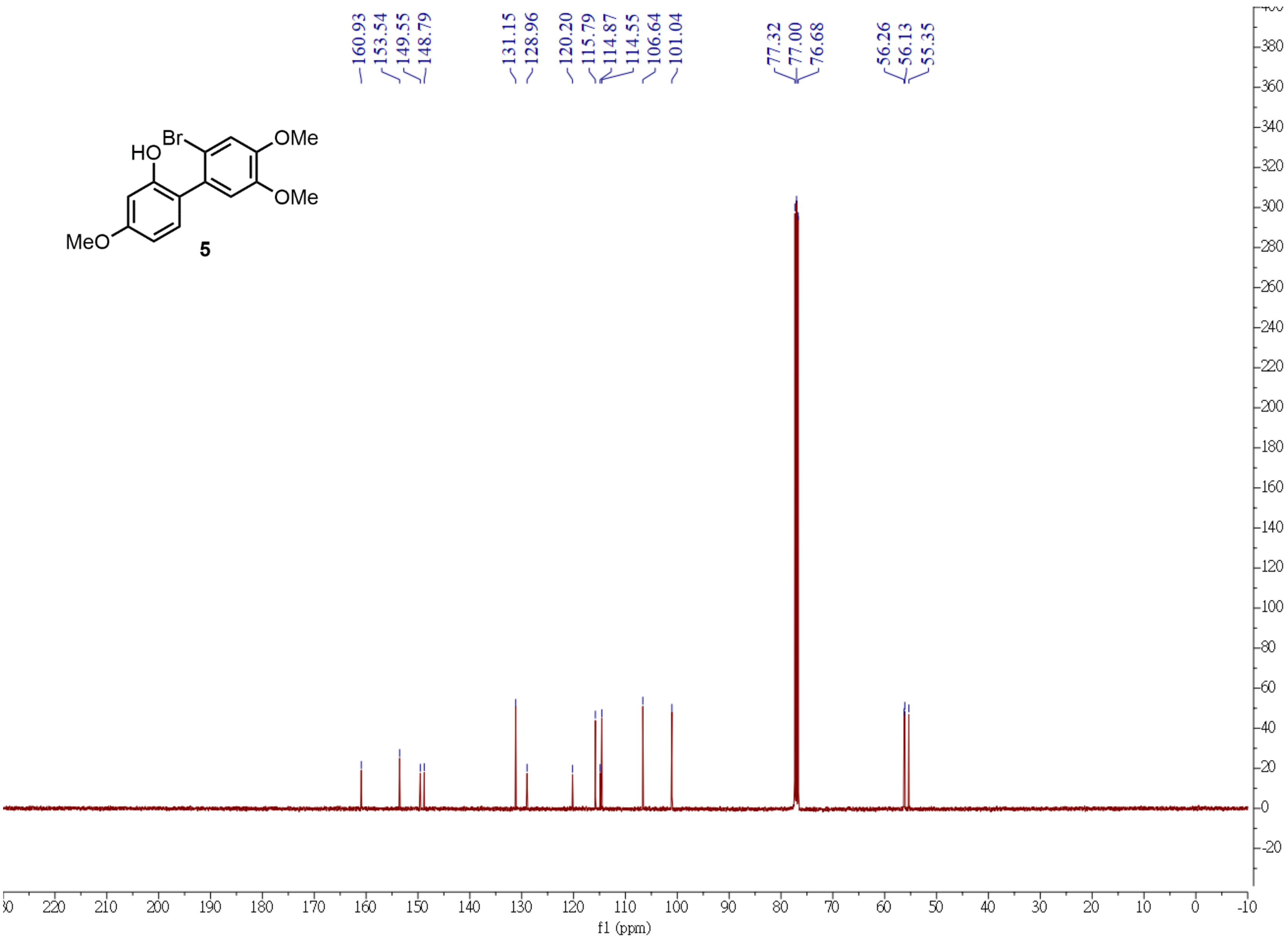

^{13}C NMR Spectrum of Compound 5 ($CDCl_3$, 100 MHz)

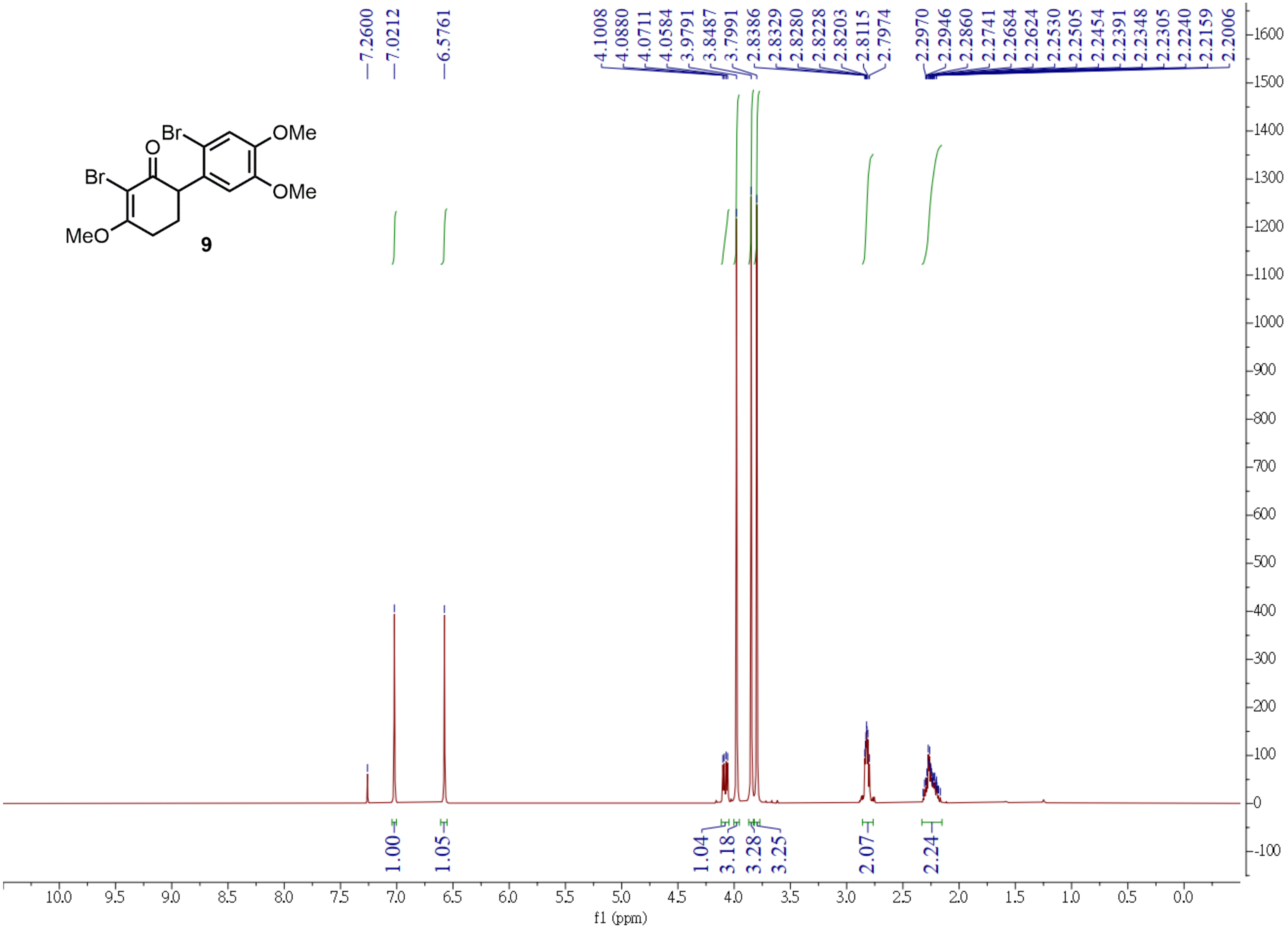

^{1}H NMR Spectrum of Compound 9 ($CDCl_3$, 400 MHz)

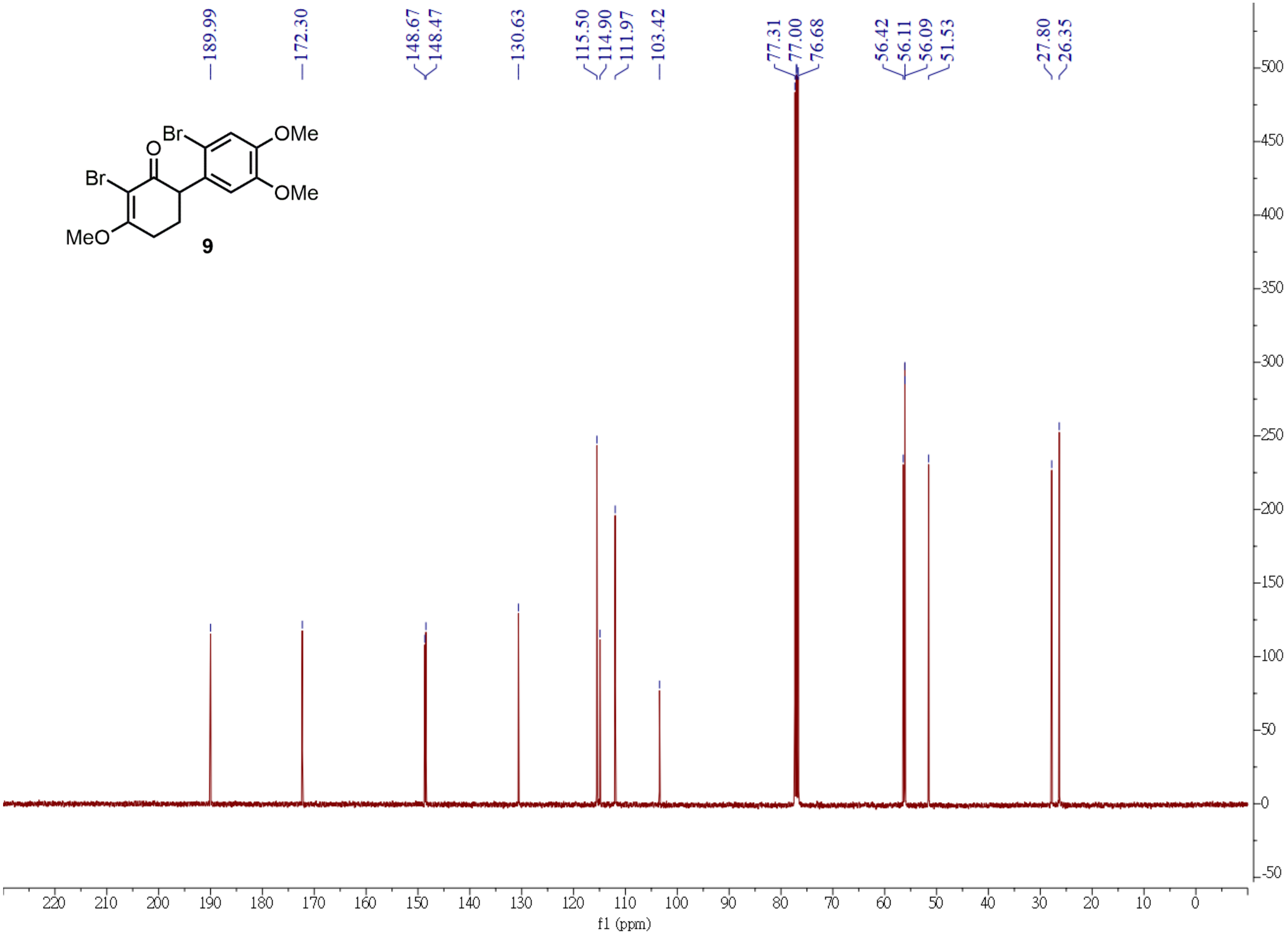

^{13}C NMR Spectrum of Compound 9 ($CDCl_3$, 100 MHz)

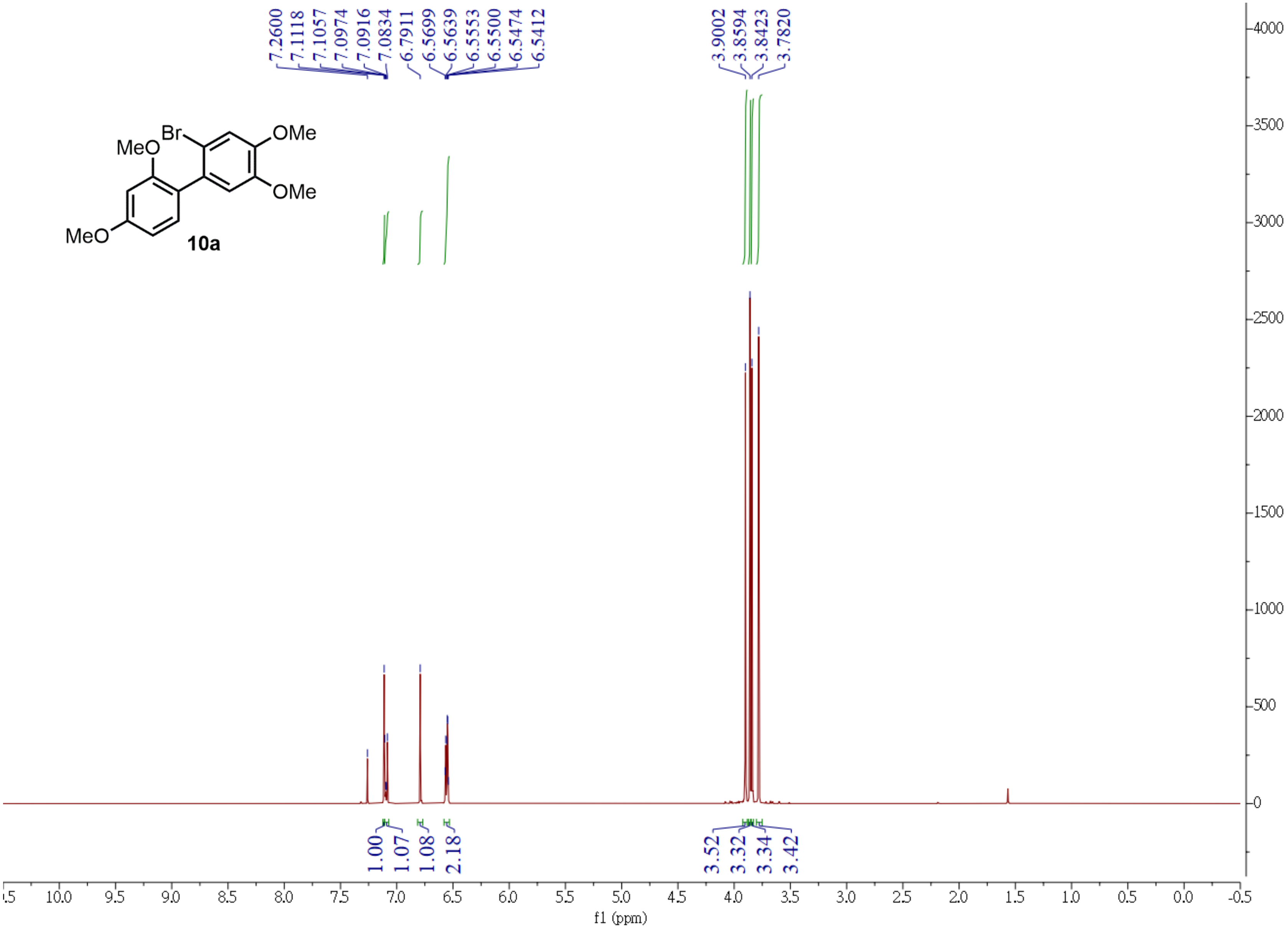

^{1}H NMR Spectrum of Compound 10a ($CDCl_3$, 400 MHz)

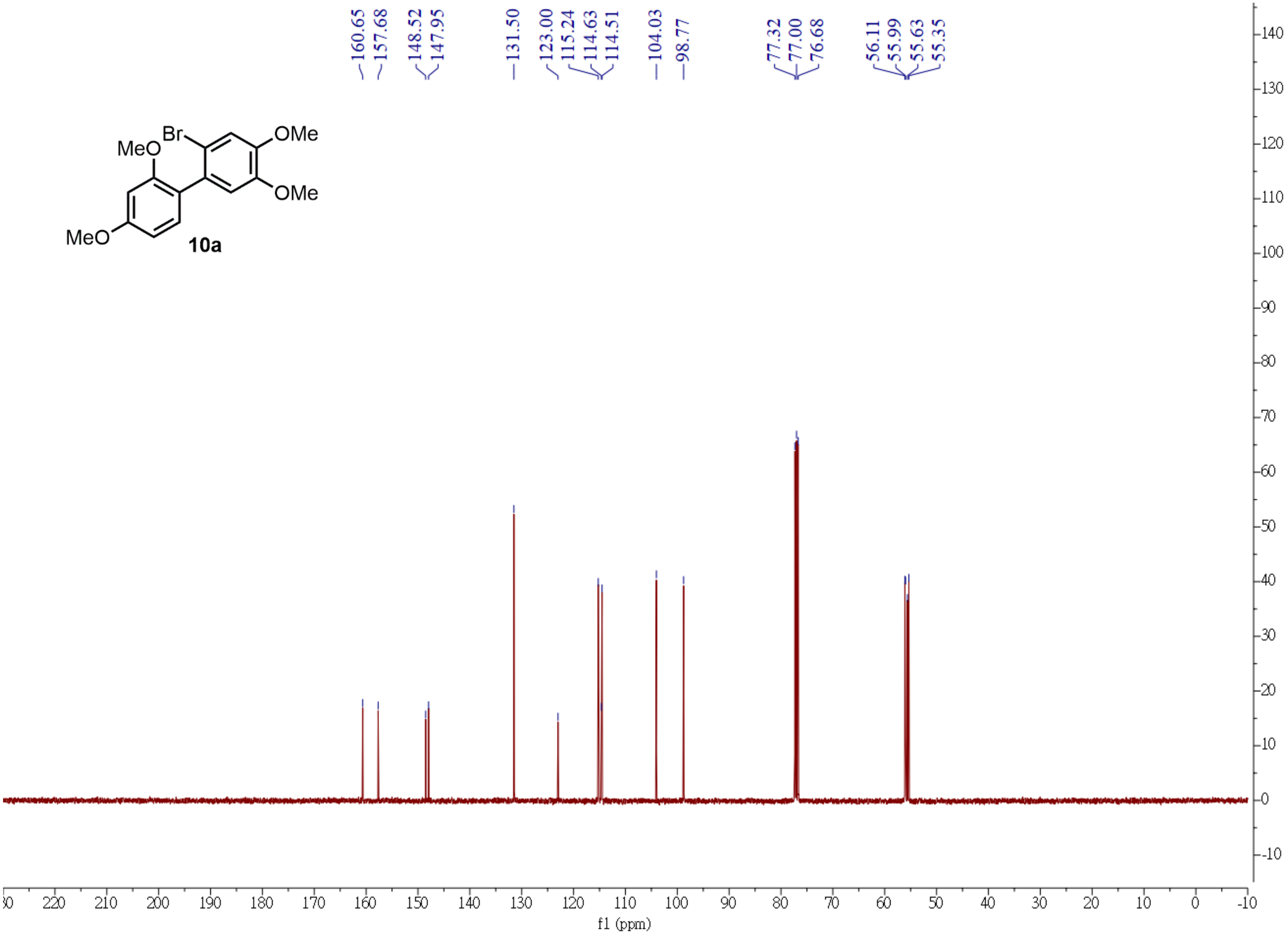

^{13}C NMR Spectrum of Compound 10a ($CDCl_3$, 100 MHz)

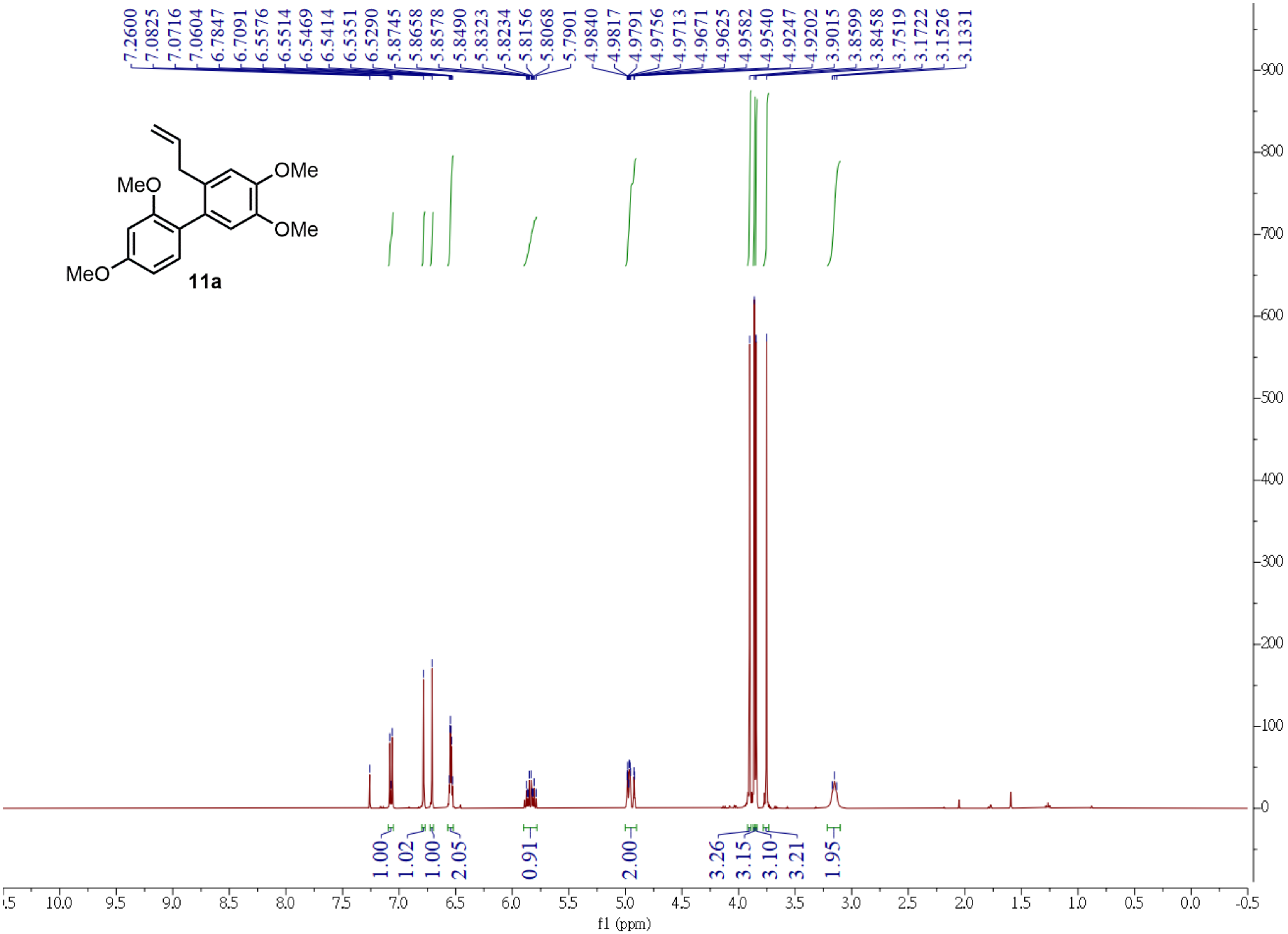

^{1}H NMR Spectrum of Compound 11a ($CDCl_3$, 400 MHz)

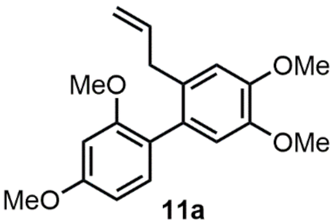

11a

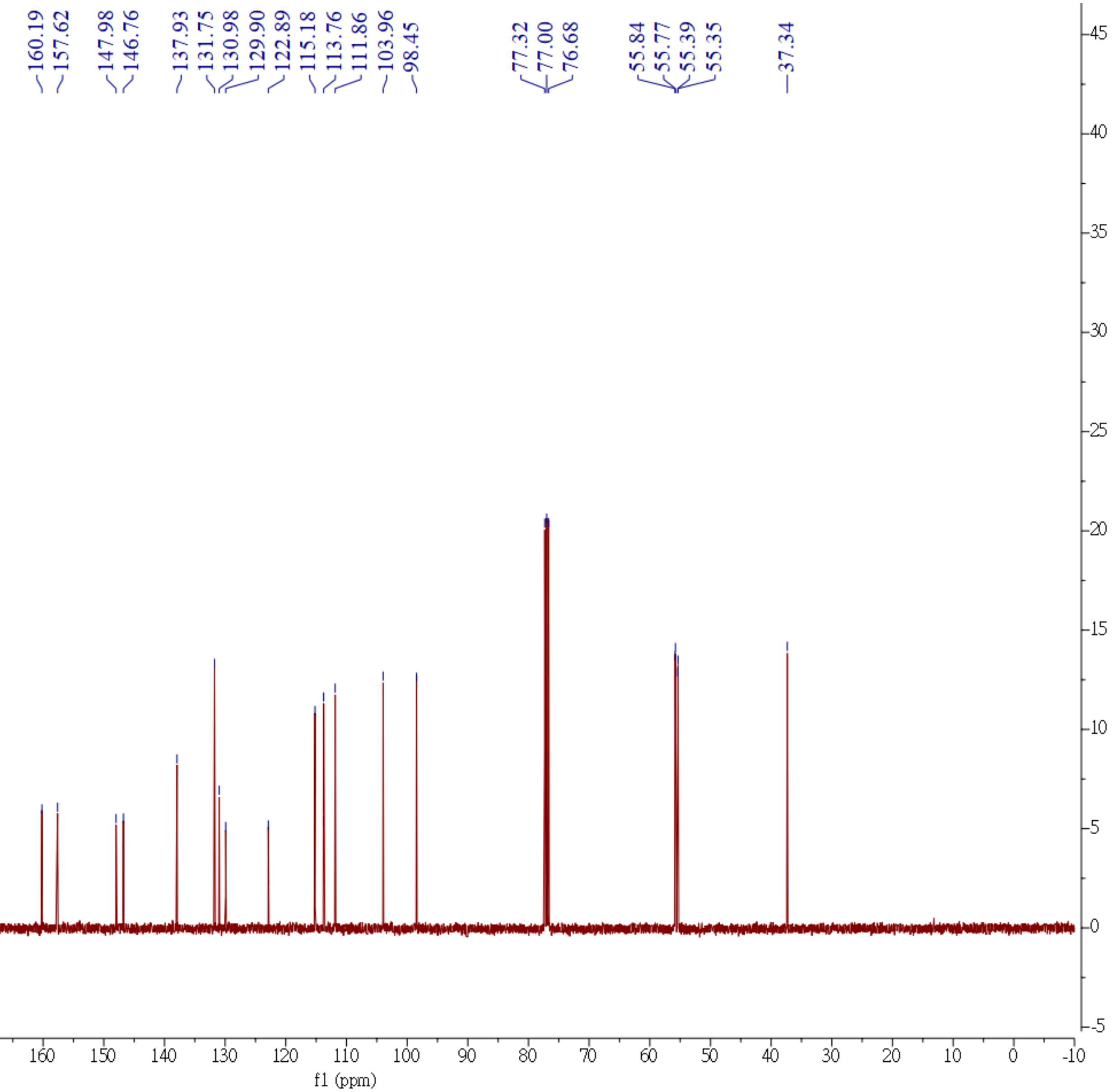

^{13}C NMR Spectrum of Compound 11a ($CDCl_3$, 100 MHz)

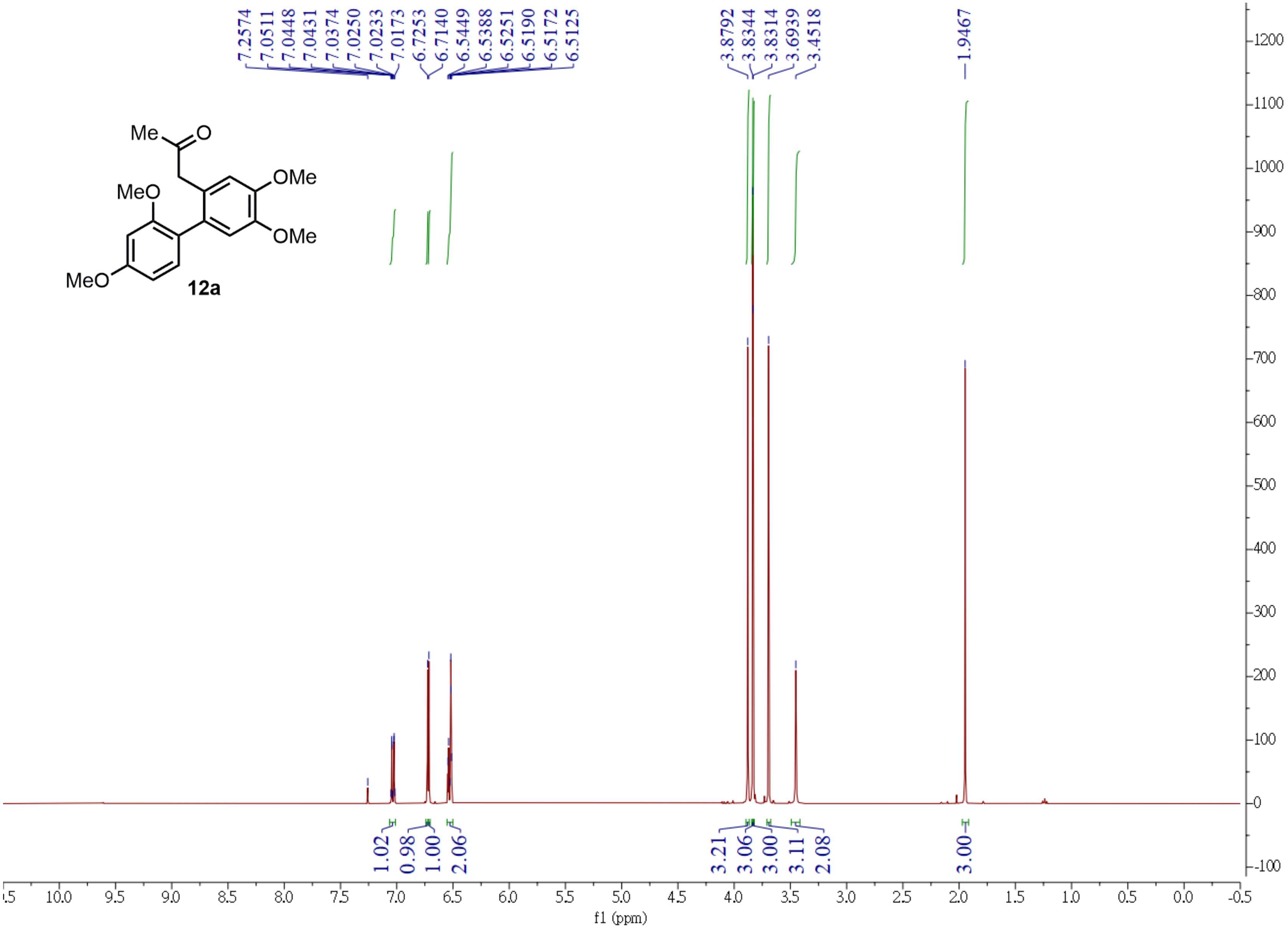

^{1}H NMR Spectrum of Compound 12a ($CDCl_3$, 400 MHz)

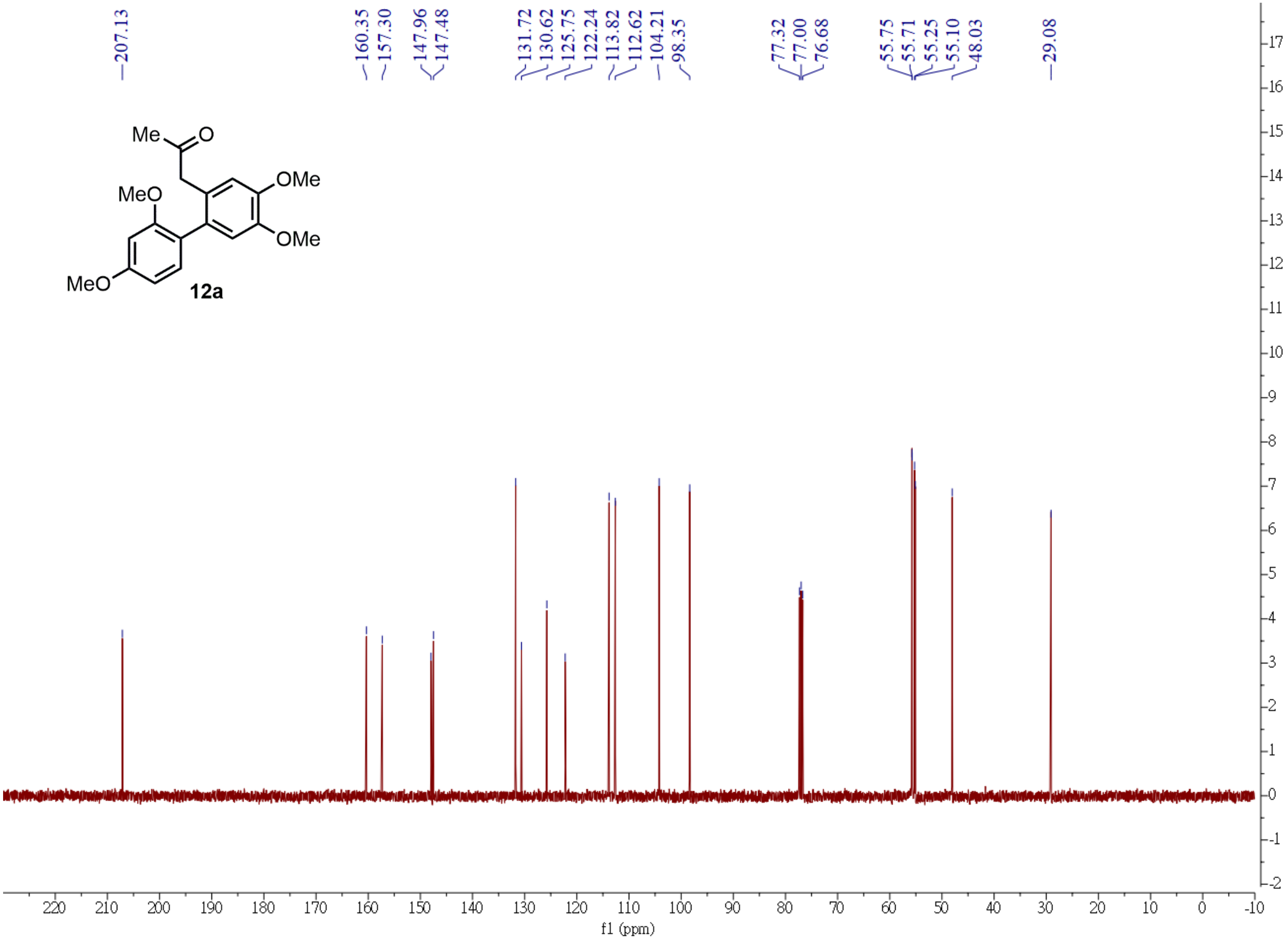

^{13}C NMR Spectrum of Compound 12a ($CDCl_3$, 100 MHz)

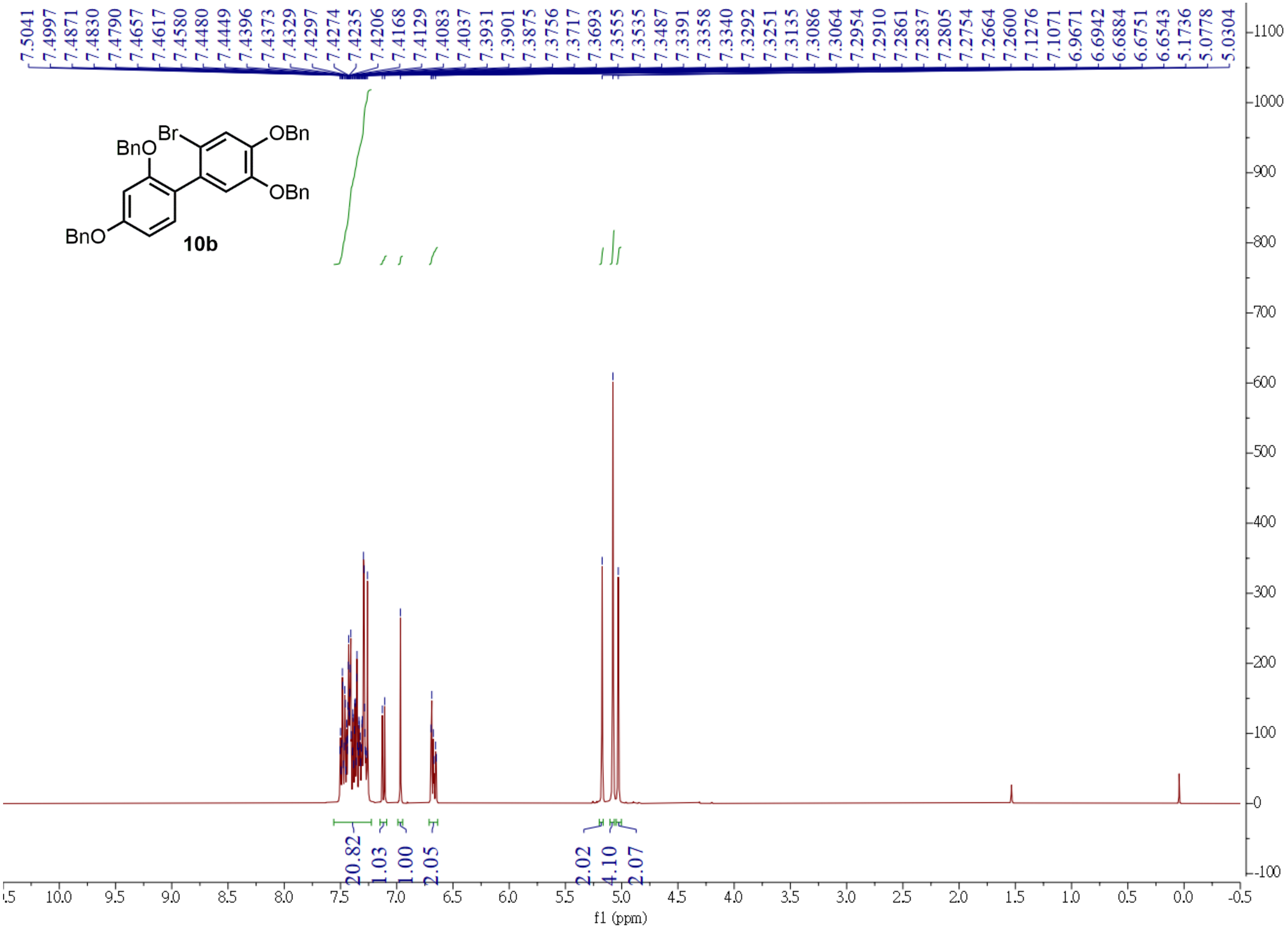

^{1}H NMR Spectrum of Compound 10b ($CDCl_3$, 400 MHz)

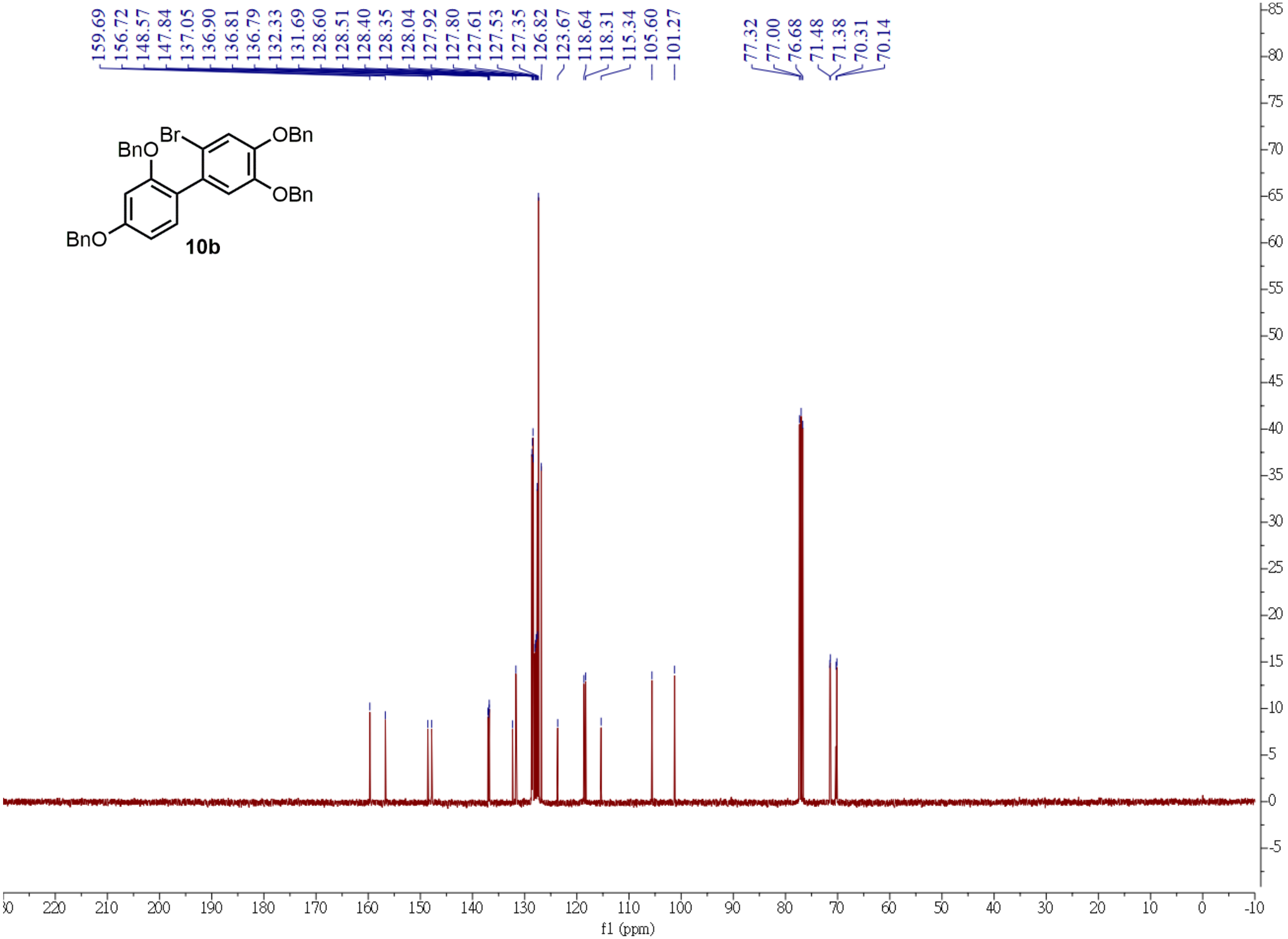

^{13}C NMR Spectrum of Compound 10b ($CDCl_3$, 100 MHz)

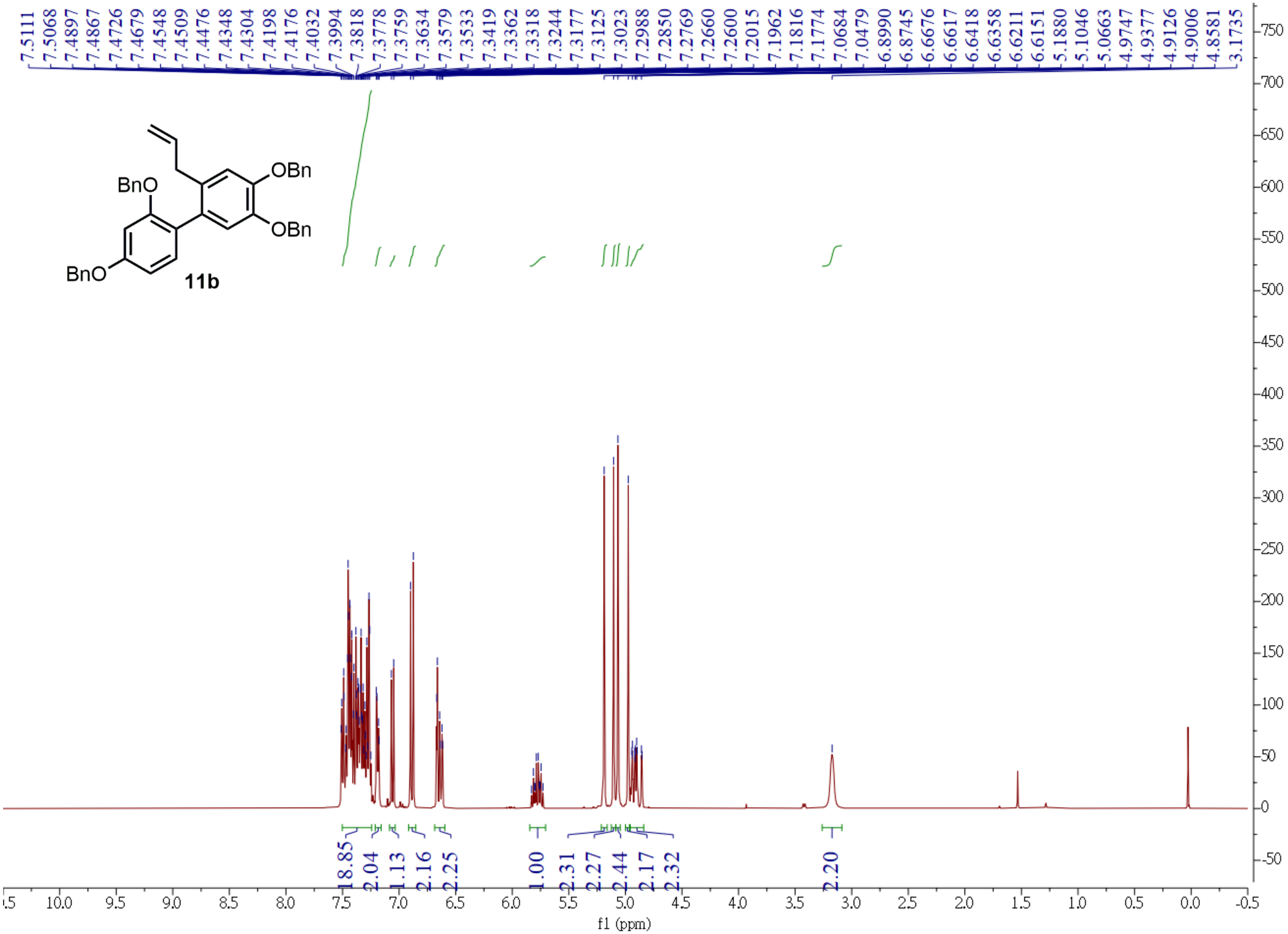

^{1}H NMR Spectrum of Compound 11b ($CDCl_3$, 400 MHz)

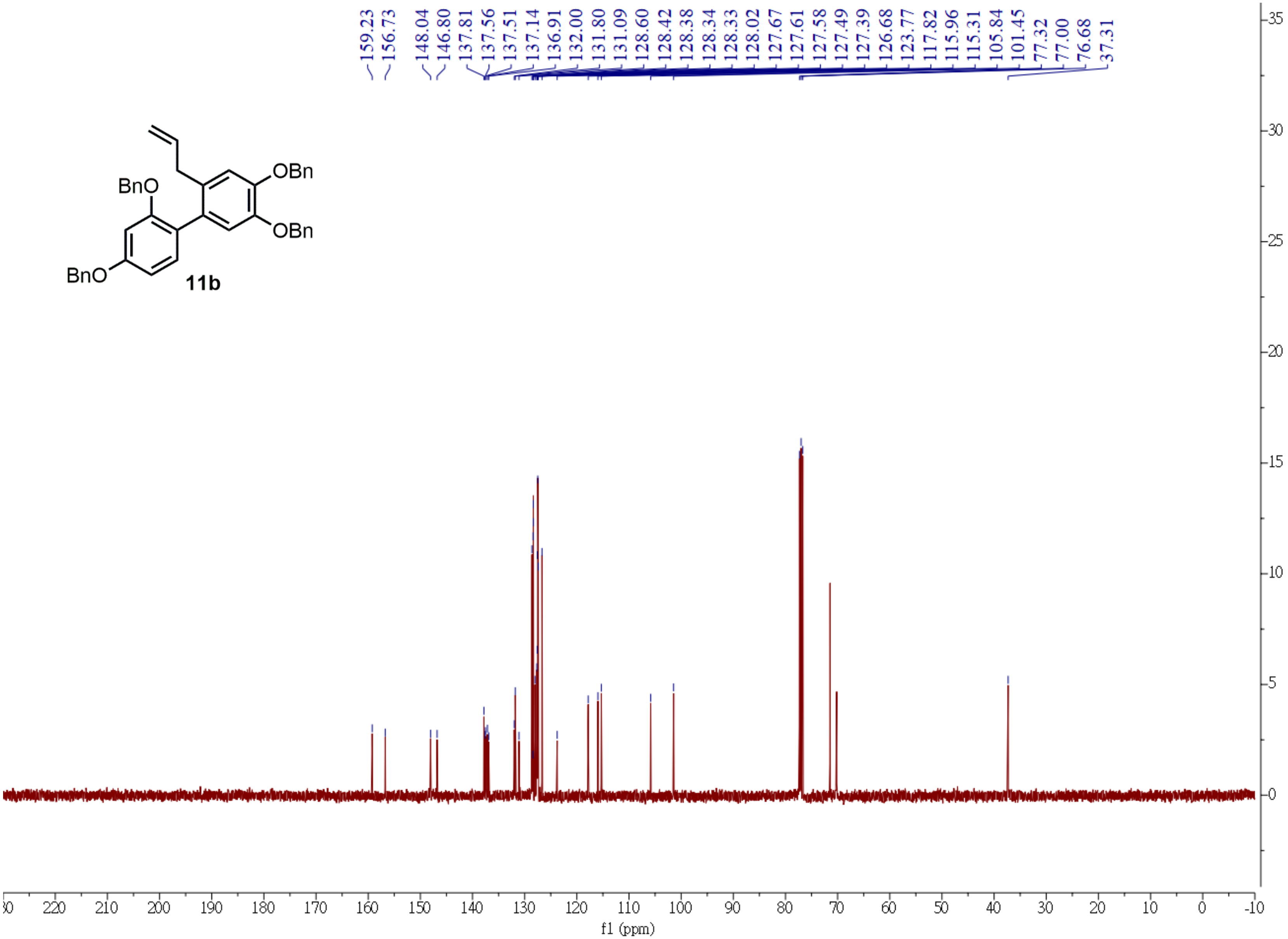

^{13}C NMR Spectrum of Compound 11b ($CDCl_3$, 100 MHz)

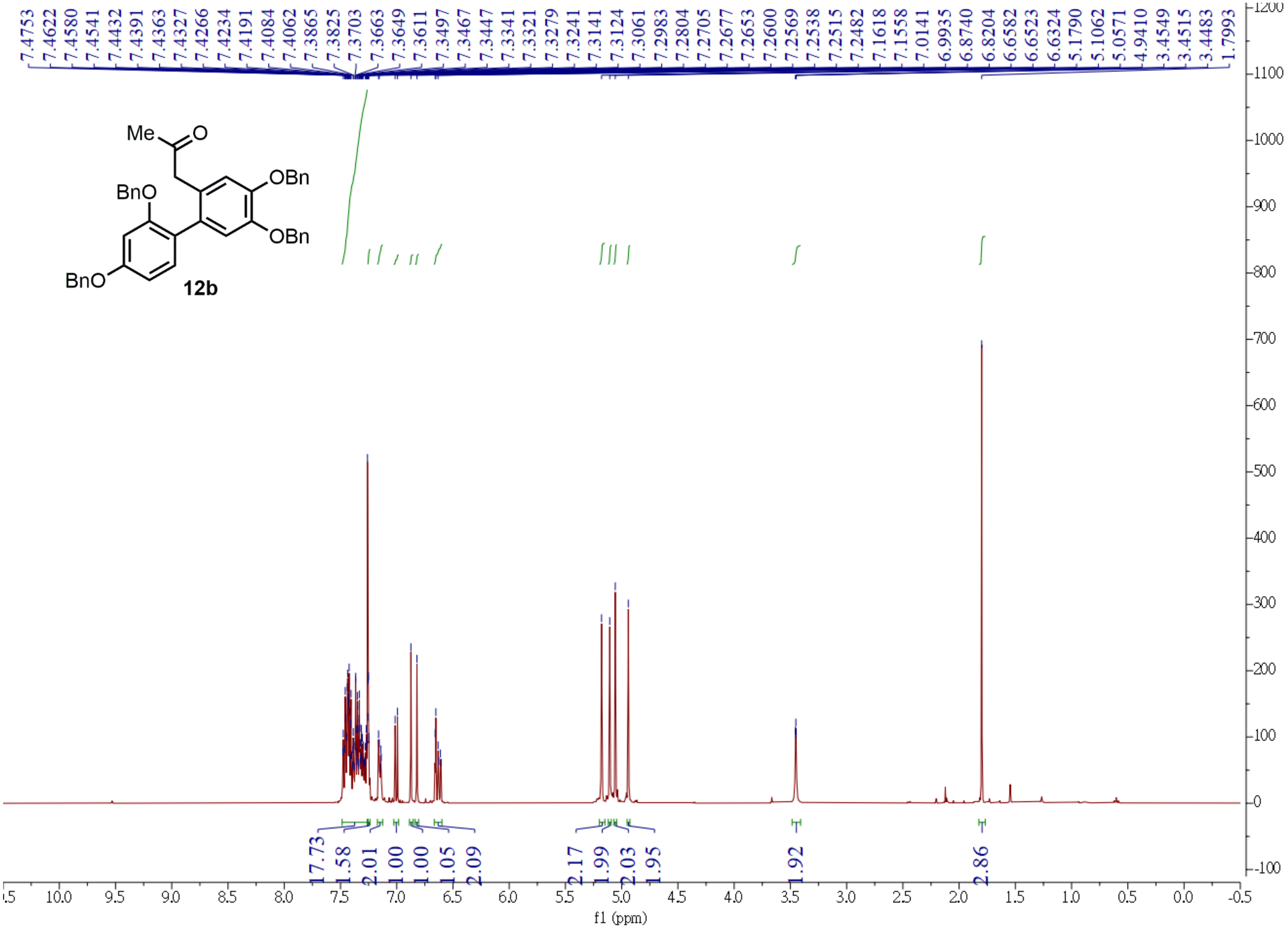

^{1}H NMR Spectrum of Compound 12b (CDCl₃, 400 MHz)

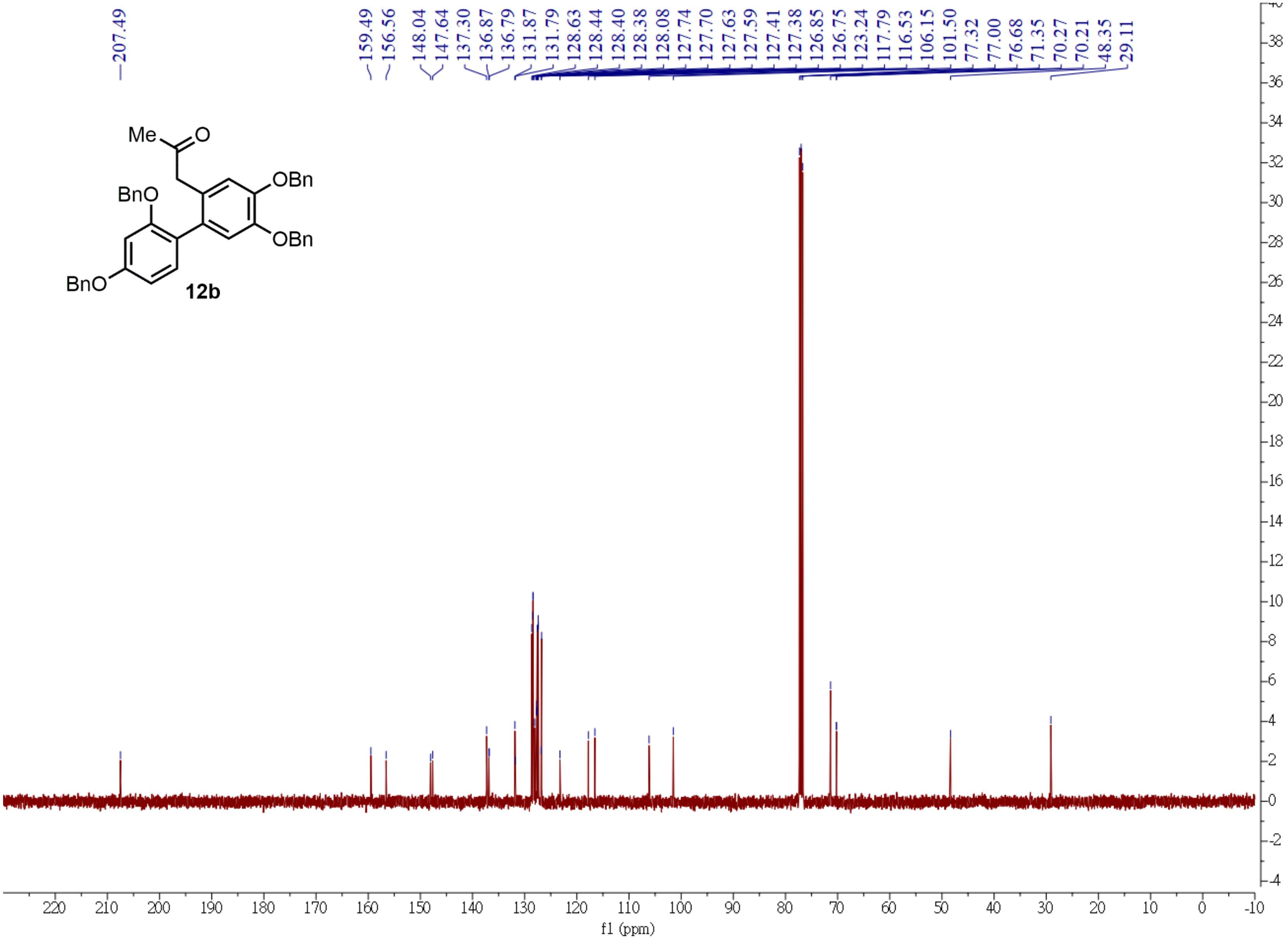

^{13}C NMR Spectrum of Compound 12b ($CDCl_3$, 100 MHz)

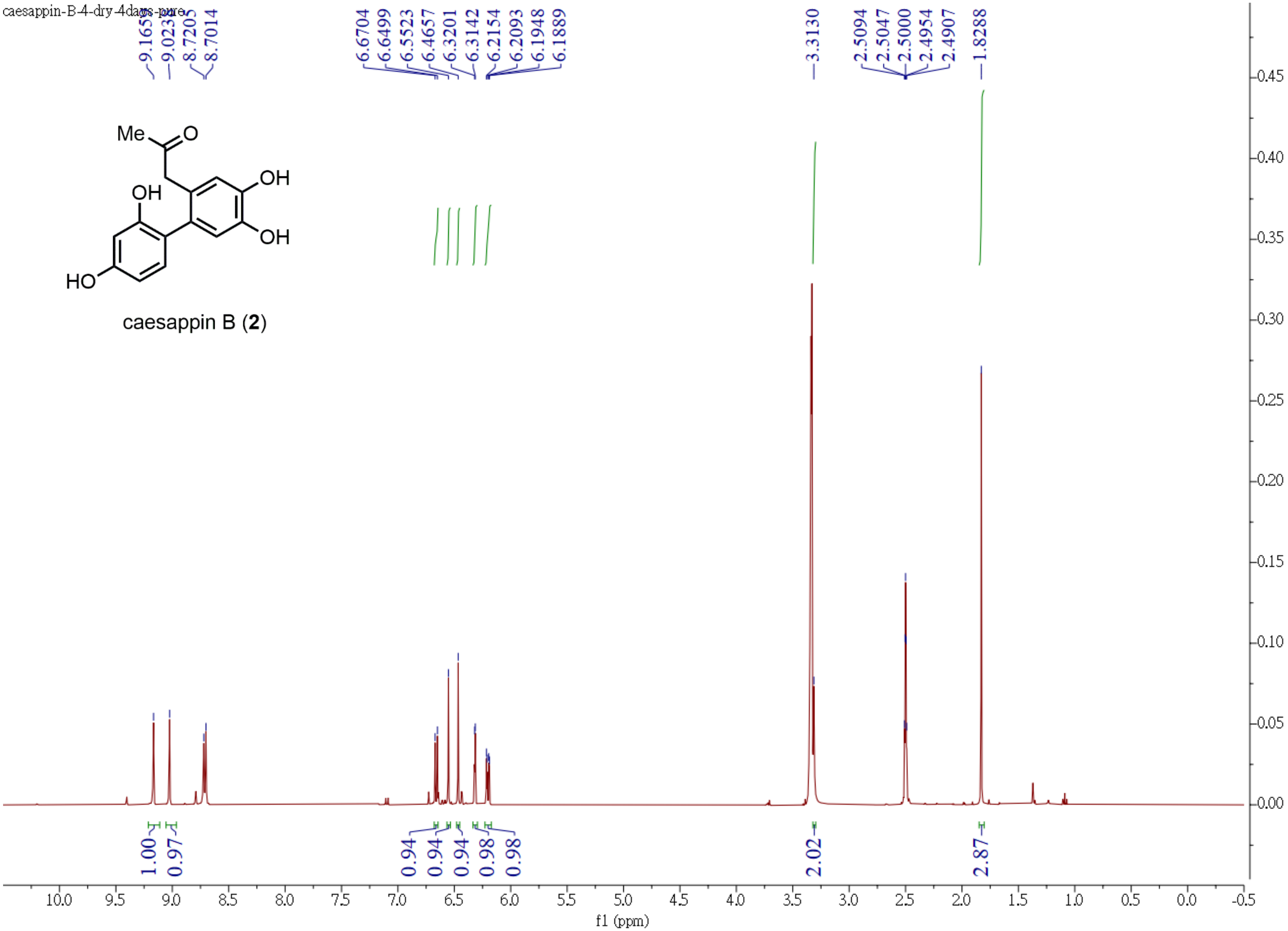

^{1}H NMR Spectrum of Caesappin B (2) (DMSO-d_6, 400 MHz)

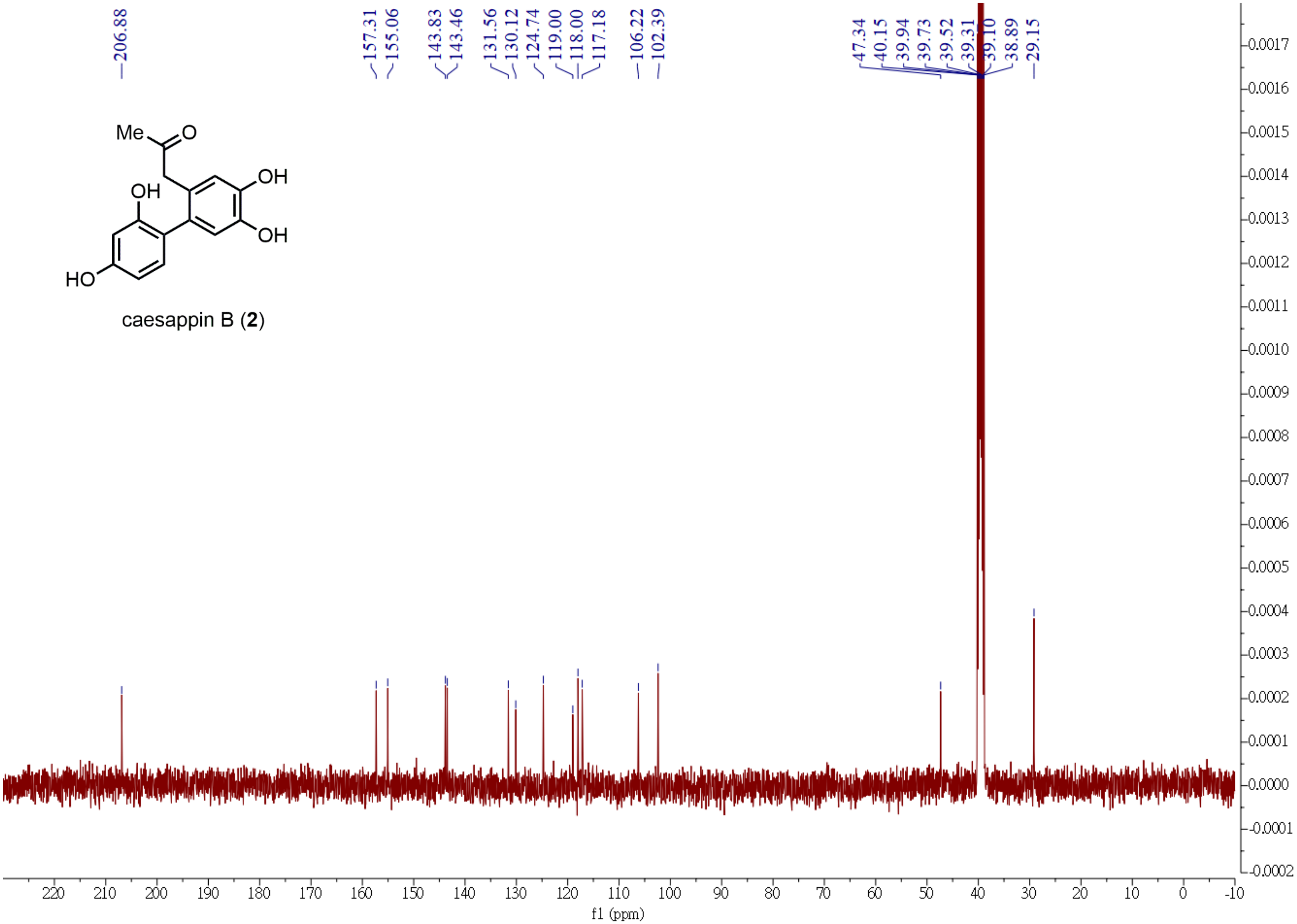

^{13}C NMR Spectrum of Caesappin B (2) (DMSO-d_6, 100 MHz)

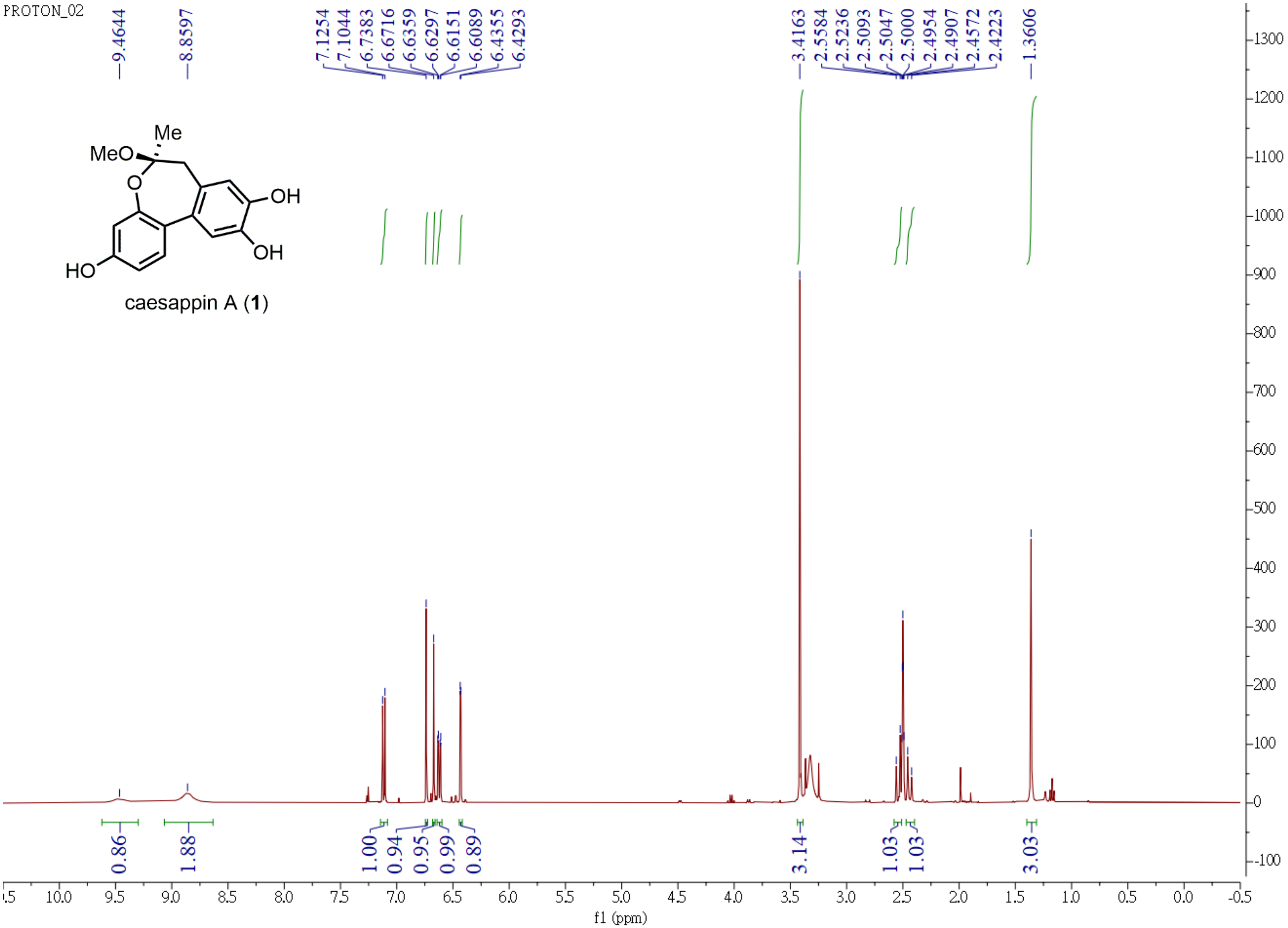

^{1}H NMR Spectrum of Caesappin A (1) (DMSO-d_6, 400 MHz)

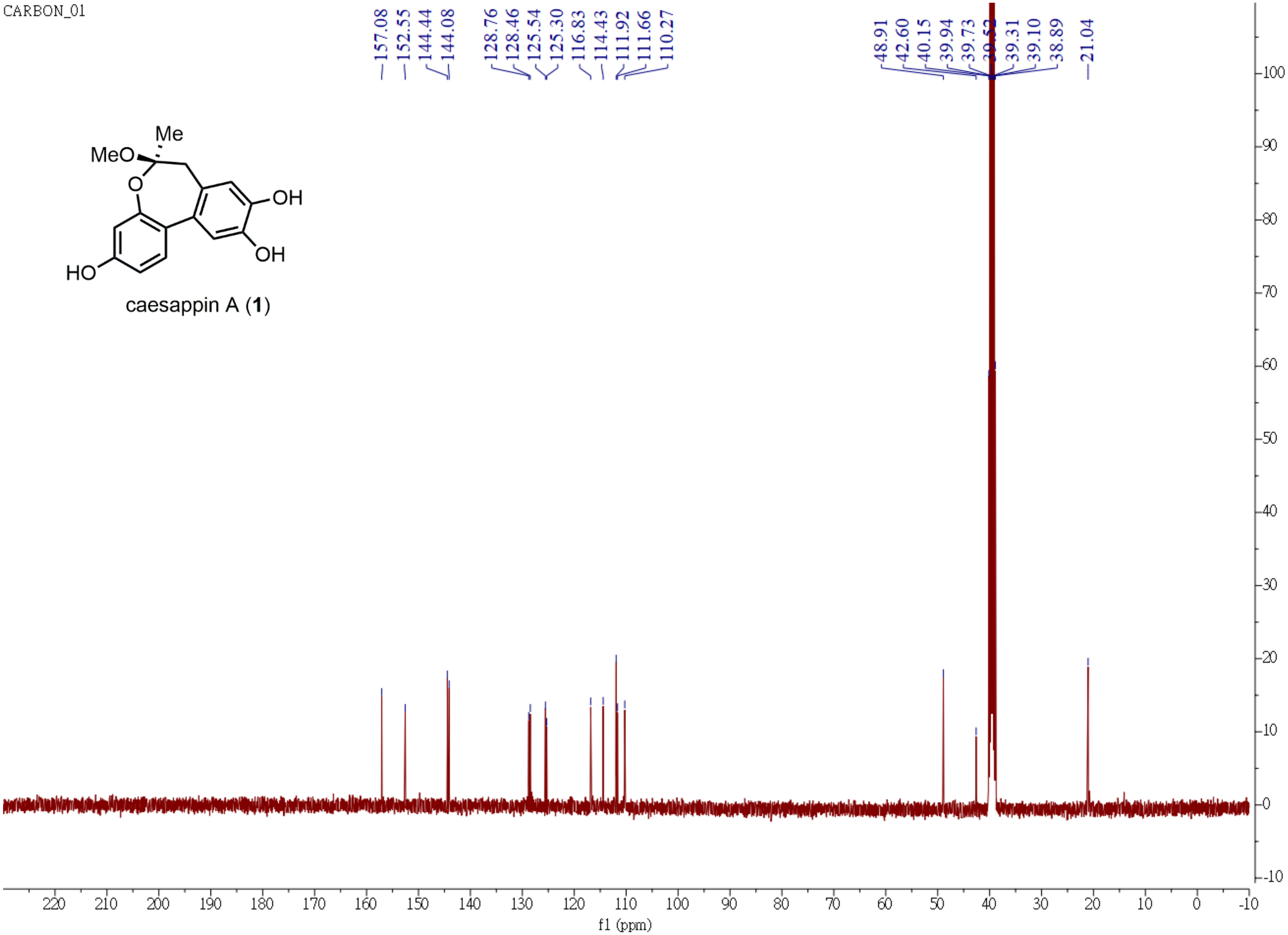

^{13}C NMR Spectrum of Caesappin A (1) (DMSO-d_6, 100 MHz)

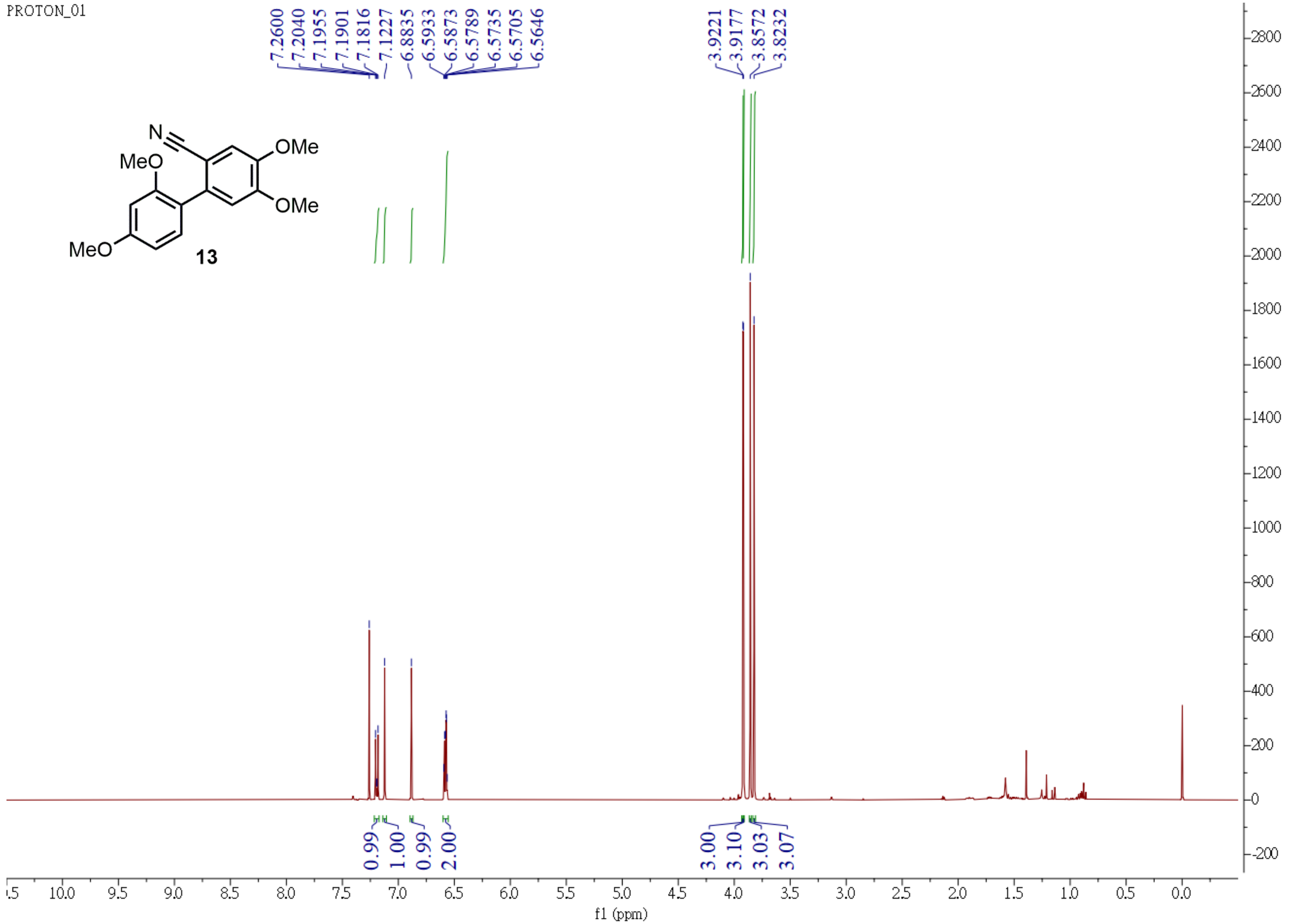

^{1}H NMR Spectrum of Compound 13 ($CDCl_3$, 400 MHz)

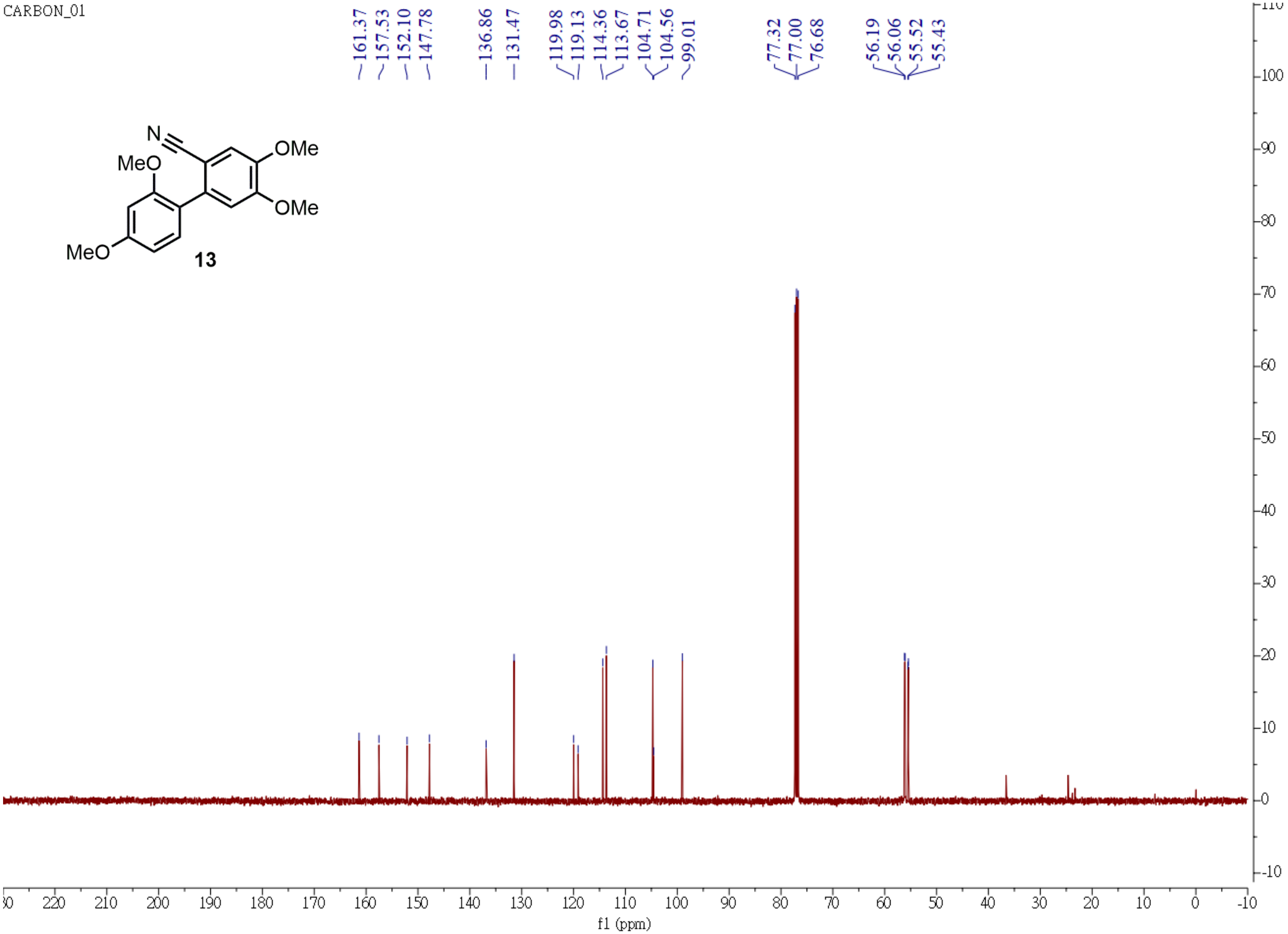

^{13}C NMR Spectrum of Compound 13 ($CDCl_3$, 100 MHz)

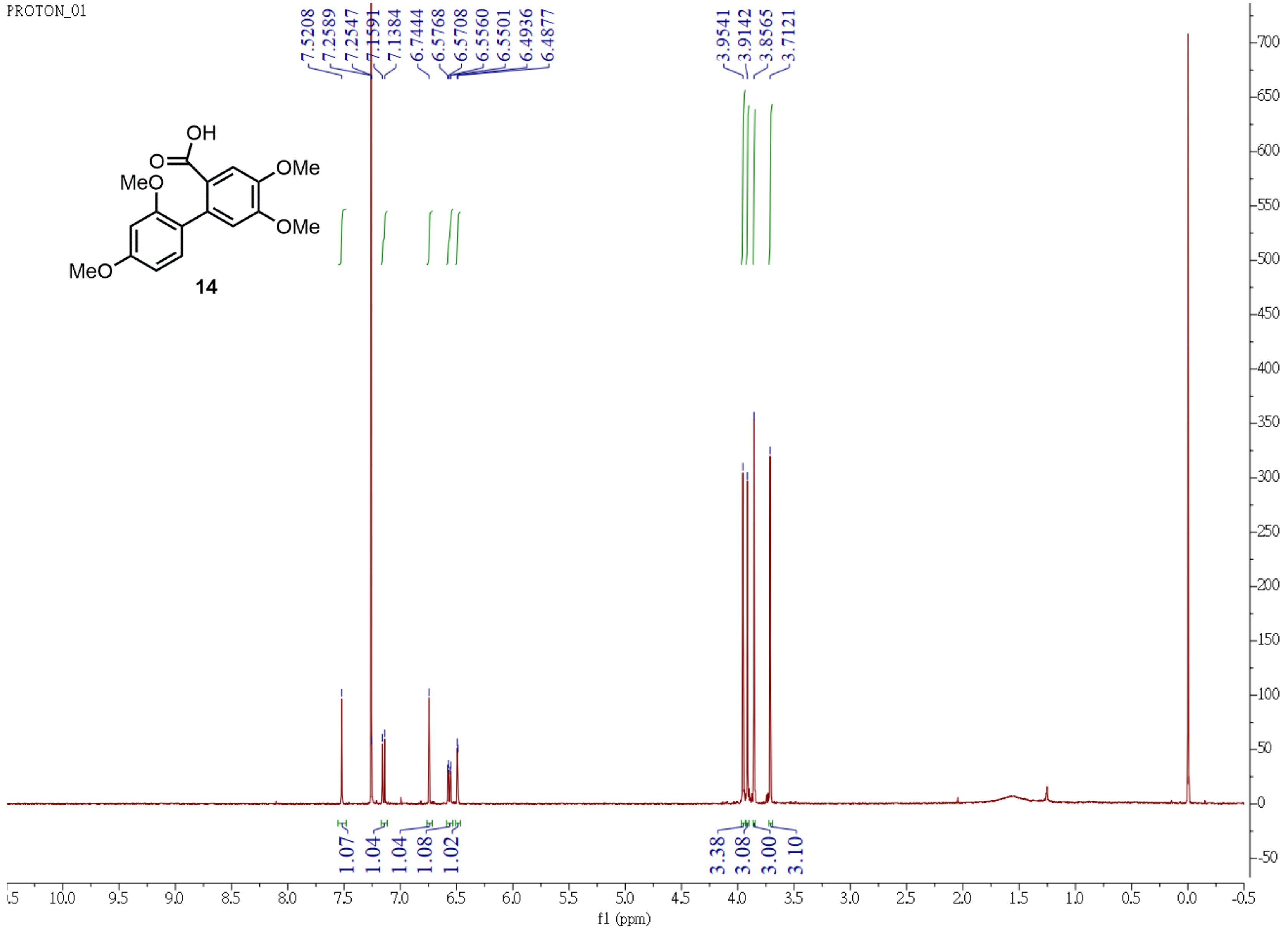

^{1}H NMR Spectrum of Compound 14 ($CDCl_3$, 400 MHz)

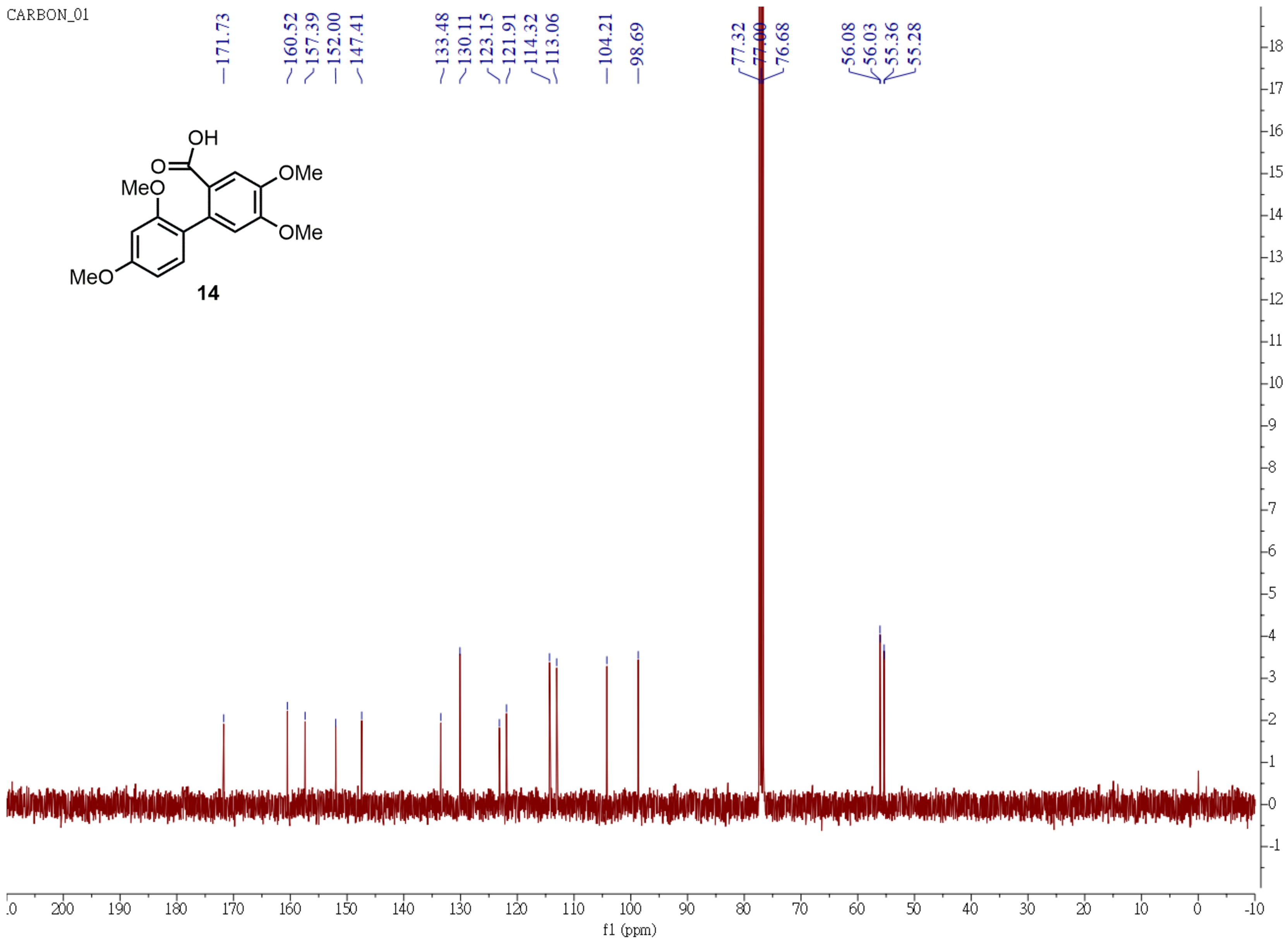

^{13}C NMR Spectrum of Compound 14 ($CDCl_3$, 100 MHz)

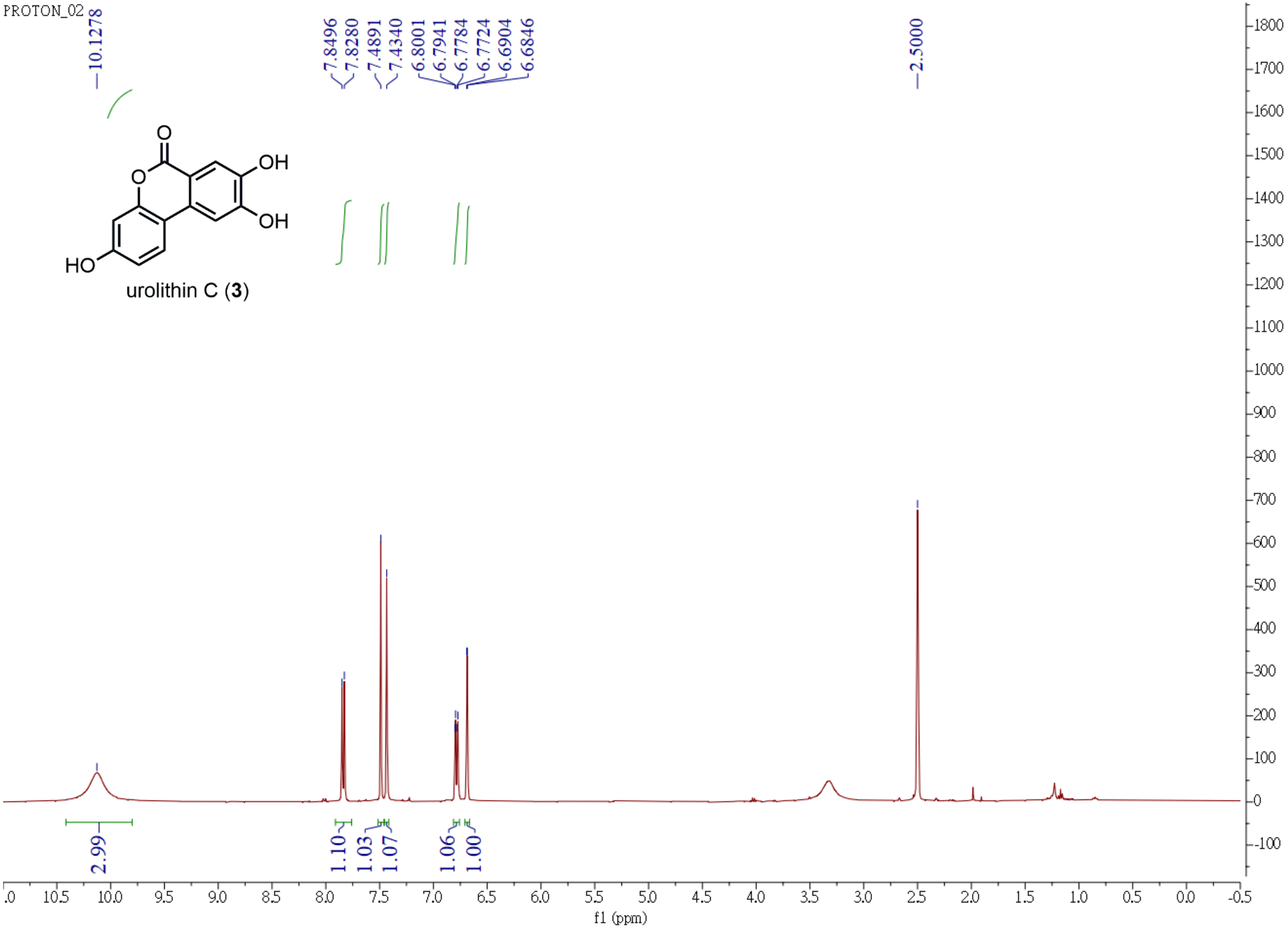

urolithin C (**3**)

^{1}H NMR Spectrum of Urolithin C (3) (DMSO-d_6, 400 MHz)

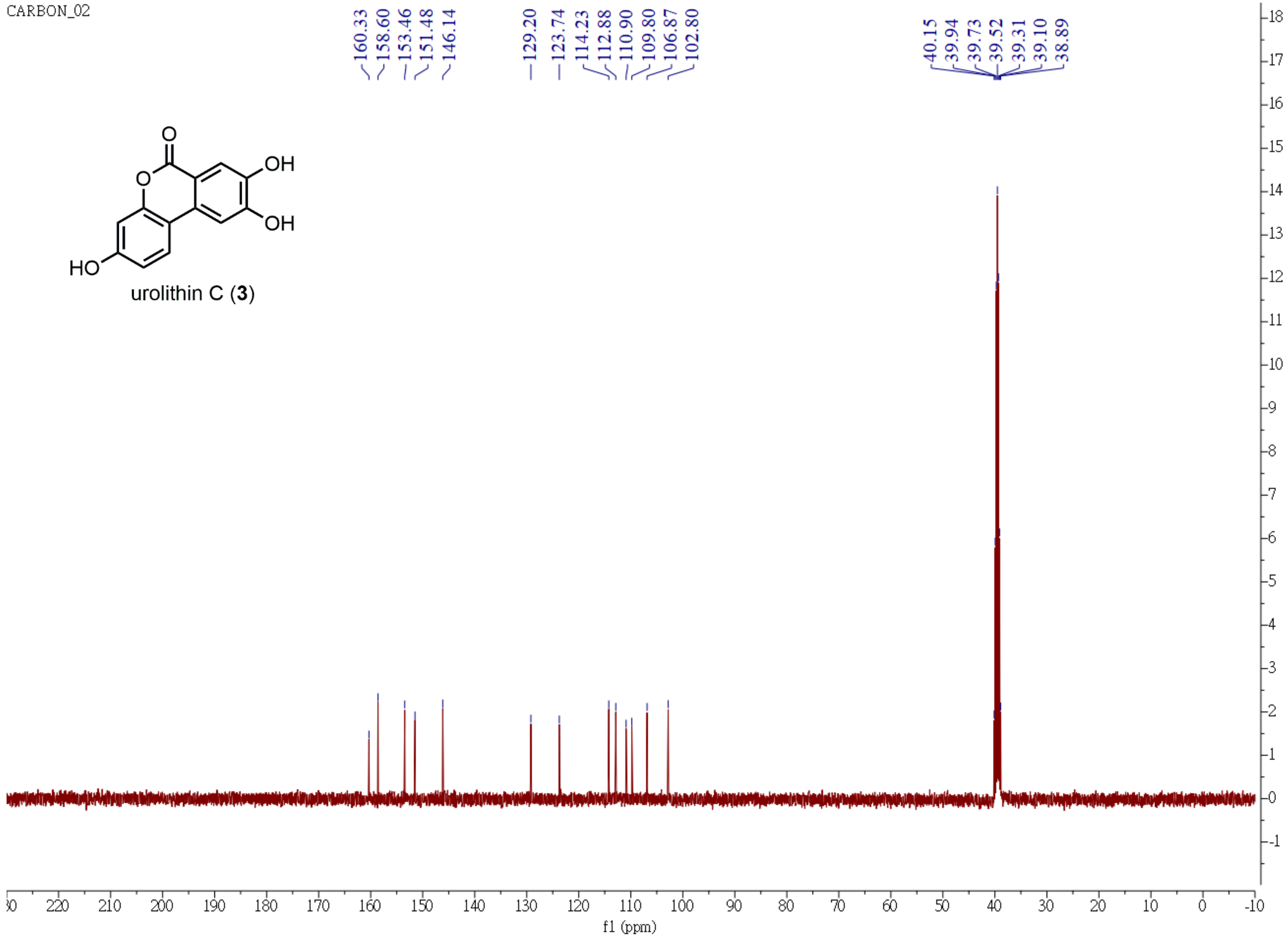

^{13}C NMR Spectrum of Urolithin C (3) (DMSO-d_6, 100 MHz)

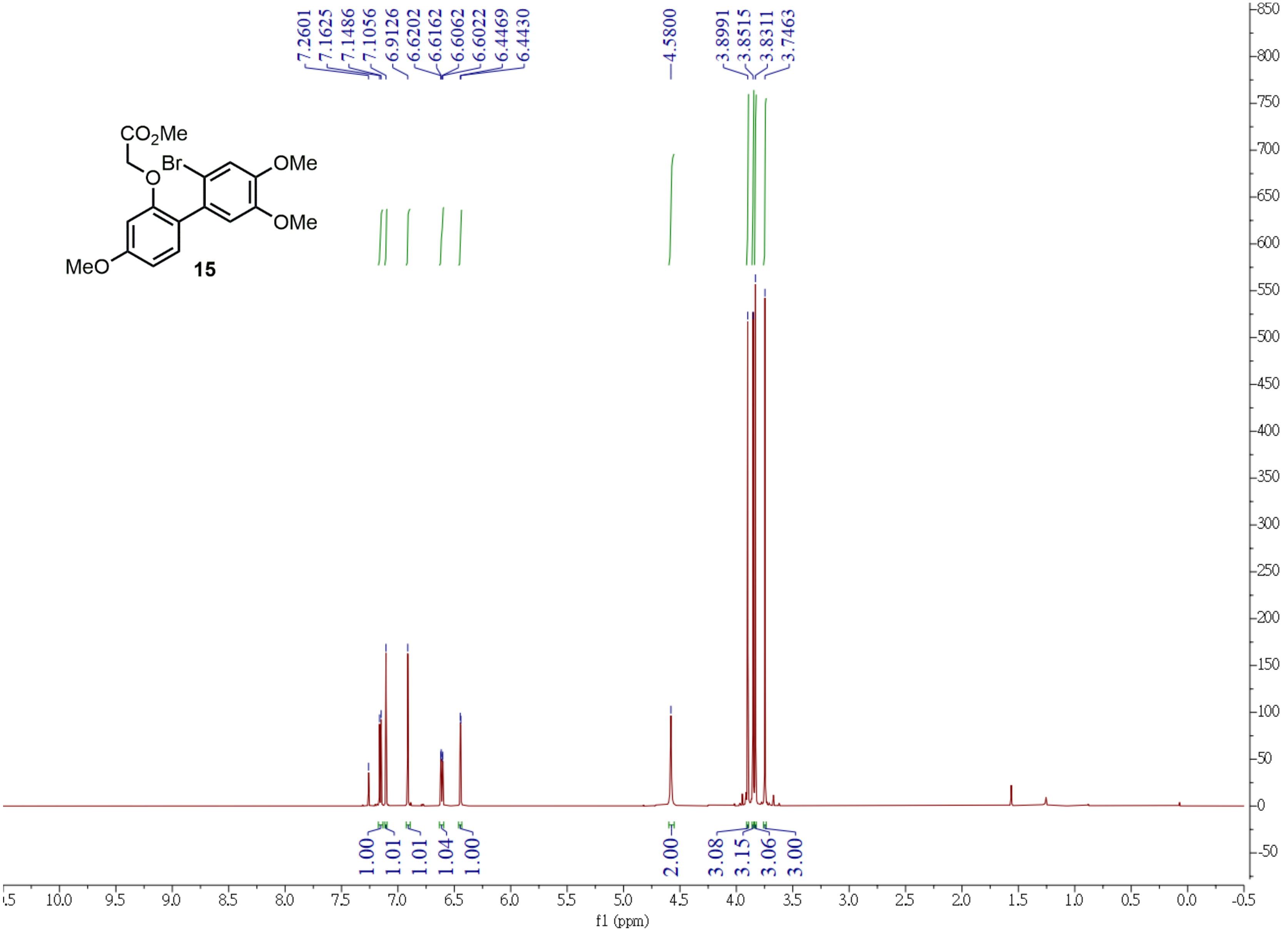

^{1}H NMR Spectrum of Compound 15 ($CDCl_3$, 400 MHz)

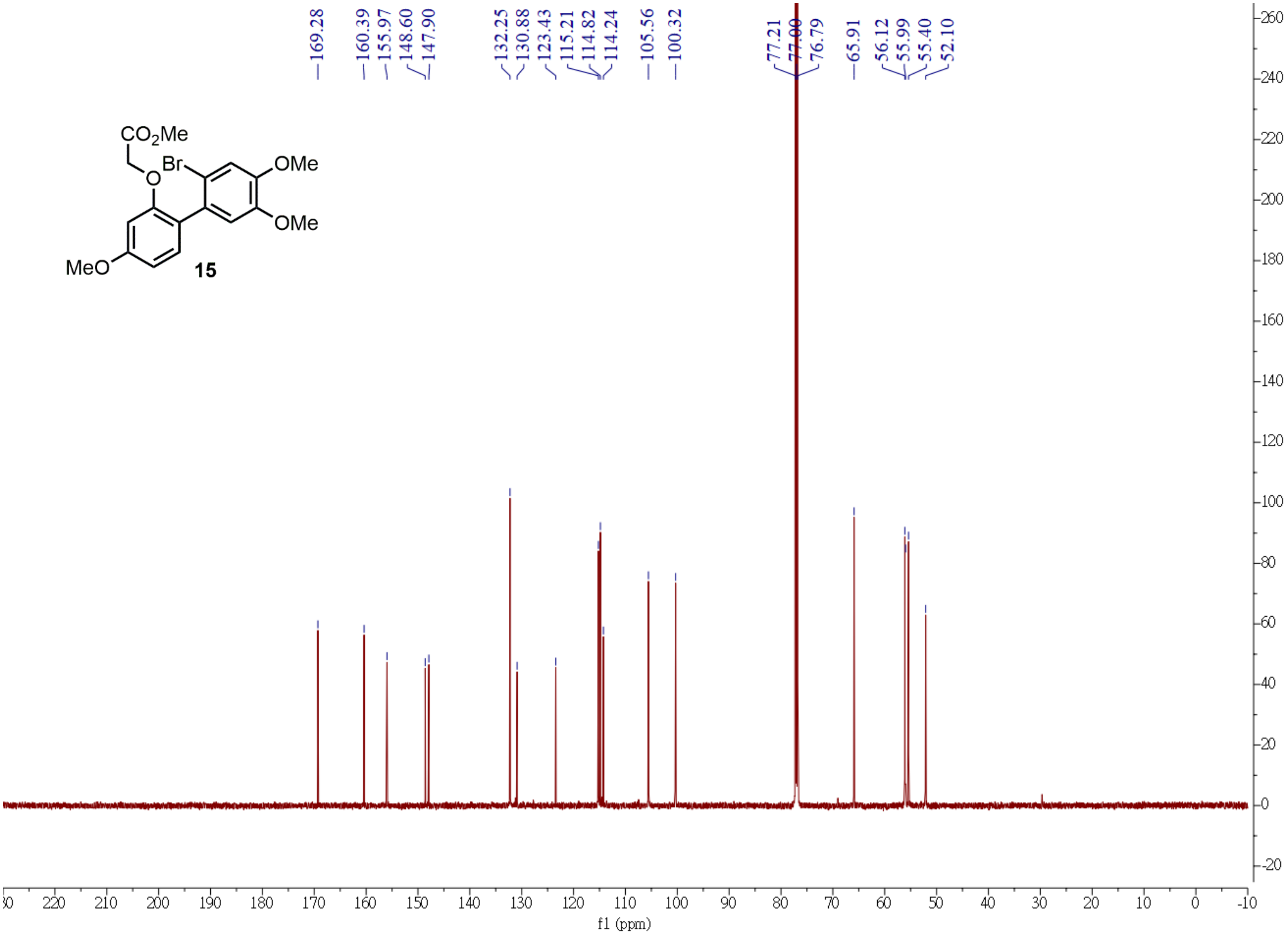

^{13}C NMR Spectrum of Compound 15 ($CDCl_3$, 100 MHz)

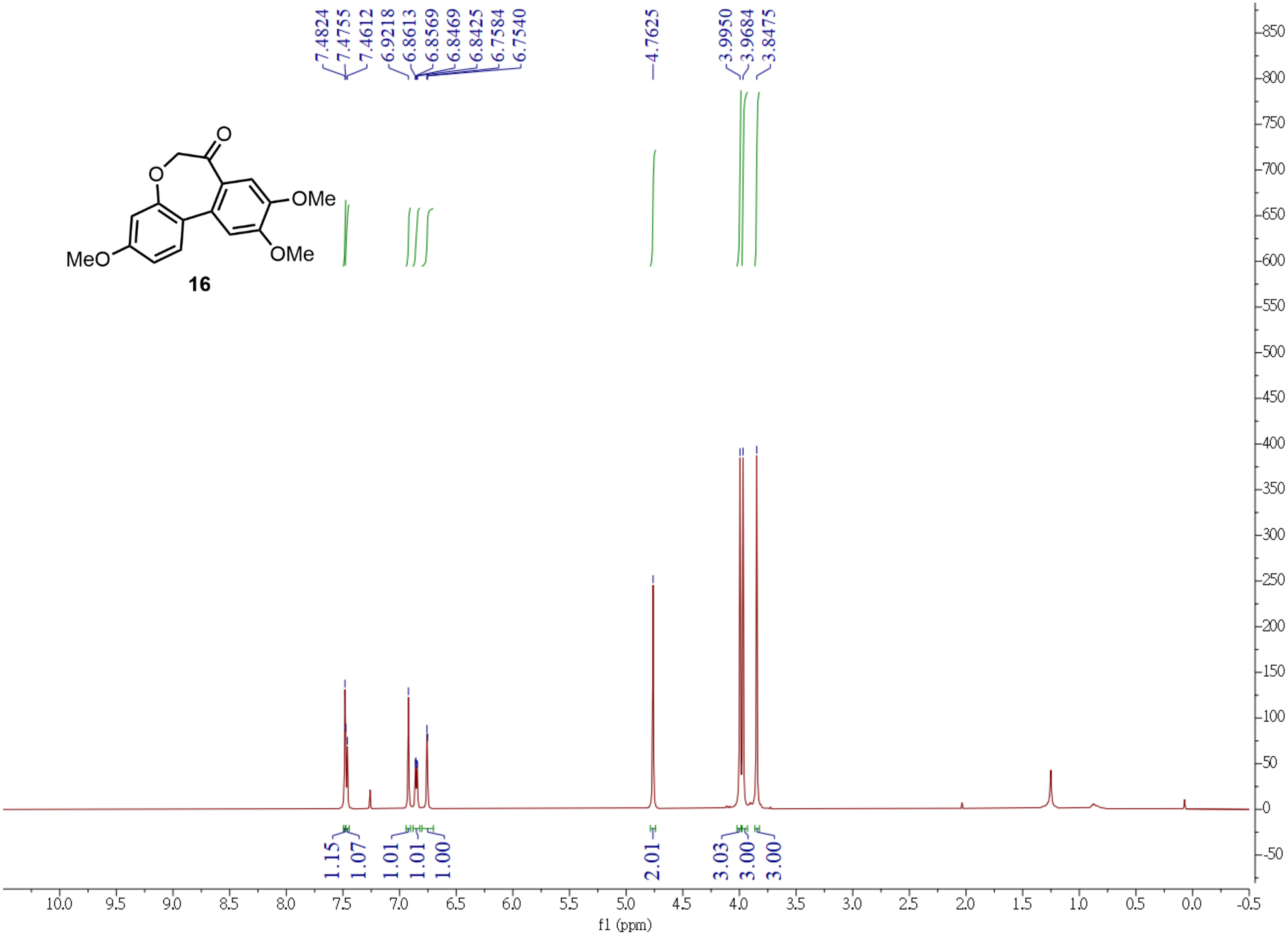

^{1}H NMR Spectrum of Compound 16 ($CDCl_3$, 400 MHz)

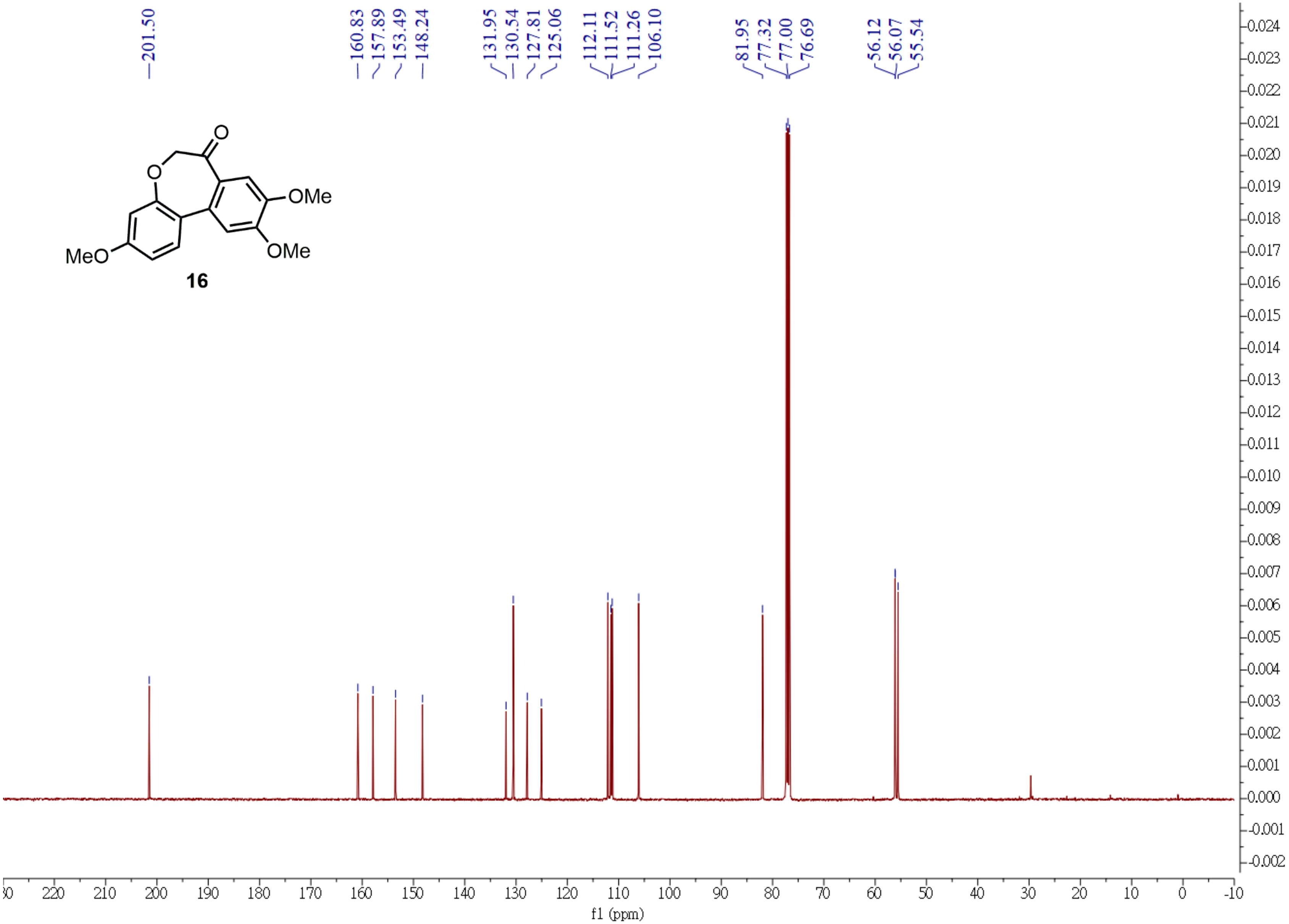

^{13}C NMR Spectrum of Compound 16 ($CDCl_3$, 100 MHz)
